# Identification of Molecular Markers That Are Specific to the Class *Thermoleophilia*

**DOI:** 10.3389/fmicb.2019.01185

**Published:** 2019-05-24

**Authors:** Danyu Hu, Yang Zang, Yingjin Mao, Beile Gao

**Affiliations:** ^1^CAS Key Laboratory of Tropical Marine Bio Resources and Ecology, Guangdong Key Laboratory of Marine Materia Medica, South China Sea Institute of Oceanology, Chinese Academy of Sciences, Guangzhou, China; ^2^University of Chinese Academy of Sciences, Beijing, China

**Keywords:** *Thermoleophilia*, phylogeny, molecular signatures, conserved signature indels, conserved signature proteins

## Abstract

The class *Thermoleophilia* is one of the deep-rooting lineages within the *Actinobacteria* phylum and metagenomic investigation of microbial diversity suggested that species associated with the class *Thermoleophilia* are abundant in hot spring and soil samples. However, very few species of this class have been cultivated and characterized. Our understanding of the phylogeny and taxonomy of *Thermoleophilia* is solely based on 16S rRNA sequence analysis of limited cultivable representatives, but no other phenotypic or genotypic characteristics are known that can clearly discriminate members of this class from the other taxonomic units within the kingdom bacteria. This study reports phylogenomic analysis for 12 sequenced members of this class and clearly resolves the interrelationship of not yet cultivated species with reconstructed genomes and known type species. Comparative genome analysis discovered 12 CSIs in different proteins and 32 CSPs that are specific to all species of this class. In addition, a large number of CSIs or CSPs were identified to be unique to certain lineages within this class. This study represents the first and most comprehensive phylogenetic analysis of the class *Thermoleophilia*, and the identified CSIs and CSPs provide valuable molecular markers for the identification and delineation of species belonging to this class or its subordinate taxa.

## Introduction

The class *Thermoleophilia* is one of the deep-rooting lineages within the *Actinobacteria* phylum and it has only recently been recognized as independent from the class *Rubrobacteria* (Zhi et al., 2009; Gao and Gupta, 2012b; Ludwig et al., 2012; Suzuki and Whitman, 2012). This class encompasses two recognized orders *Thermoleophilales* and *Solirubrobacterales* according to the most updated *Bergey’s Manual of Systematics of Archaea and Bacteria* (Suzuki and Whitman, 2015). A deep branching order *Gaiellales* within the phylum *Actinobacteria* (Albuquerque et al., 2011) has been proposed as an order of this class based on phylogenetic position, signature nucleotides of 16S rRNA, and physicochemical characteristics (Foesel et al., 2016). However, only one type strain *Gaiella occulta* F2-233 from this order was included in the analyses and its position in the phylogenetic tree is between the boundary of other *Thermoleophilia* orders and *Rubrobacteria*. The order *Thermoleophilales* only contains one family *Thermoleophilaceae* with a single genus *Thermoleophilum*. Species of this genus are small regular rods, moderately thermophilic, and obligately aerobic (Suzuki and Whitman, 2012). Their distinct feature is growth restriction to substrate n-alkanes (Zarilla and Perry, 1986), thus these species are named as heat- and oil-loving microbes, “*Thermoleophilum.*” While *Thermoleophilum* species are generally isolated from hot springs, members of the second order *Solirubrobacterales* are mainly detected in soil samples, and they exhibit more species diversity and different phenotypic characteristics. According to the most updated description of the taxonomic framework of the *Actinobacteria* phylum (Salam et al., 2019), the order *Solirubrobacterales* is composed of four families including *Solirubacteraceae*, *Conexibacteraceae*, *Parviterribacteraceae* and *Patulibacteraceae*. Currently described species of this order are mostly mesophilic with some psychrotolerant (Suzuki and Whitman, 2012). For example, metagenomic surveys of microbial diversity of soil samples from Antarctica revealed the presence of *Thermoleophilia* organisms, which can reach 15% abundance in some samples (Ji et al., 2016; Pulschen et al., 2017). Moreover, their preferred carbon sources are more diverse, including complex proteinaceous substrates, many sugars and a few other compounds (Foesel et al., 2016).

Several microbial diversity investigations suggest that *Thermoleophilia* species are abundant and diverse in nature (Joseph et al., 2003; Janssen, 2006), and they play an important role in geochemical recycling (Almeida et al., 2013; Ji et al., 2017; Li et al., 2018). However, similar to other deep-rooting classes with the phylum *Actinobacteria*, such as *Acidimicrobiia*, *Rubrobacteria*, *Nitriliruptoria*, etc., the cultivated isolates of *Thermoleophilia* are very limited (Ludwig et al., 2012; Suzuki and Whitman, 2015). Therefore, phenotypic characteristic descriptions of higher taxonomic ranks (e.g., class, order, family, and genus) within these classes are either lacking or speculative, which may not represent other yet uncultivated members belonging to these groups. In addition, our understanding of the phylogeny or taxonomy of the class *Thermoleophilia* is solely based on 16S rRNA sequence analysis, including their branching patterns in the phylogenetic trees or taxon-specific 16S rRNA signature nucleotides (Foesel et al., 2016; Salam et al., 2019). Except these two standards, no other molecular, biochemical or physiological characteristics are known that can clearly distinguish *Thermoleophilia* species from other *Actinobacteria*. Consequently, the bioprospecting or utilization of this group of bacteria is limited by our lack of knowledge of them. In the recent years, efforts have been made such as the “Genomic Encyclopedia of Bacteria and Archaea” (GEBA) project to sequence a diverse collection of the underrepresented phylogenetic lineages (Mukherjee et al., 2017), or to reconstruct genomes from metagenomic data for not yet cultivated species (Parks et al., 2017; Cabello-Yeves et al., 2018; Woodcroft et al., 2018). At the time of January 2018, there are 6 complete genomes and 10 genome assemblies for the class *Thermoleophilia*, providing great resource to explore phenotypic and genomic features of these microbes.

Two kinds of molecular markers have been described to define or delineate different higher taxa (e.g., genus level and above) for different prokaryotic phyla (Gupta and Gao, 2010; Gao and Gupta, 2012a). One kind of these molecular markers are conserved signature indels (CSIs) that are uniquely found in the genes/proteins homologs of a certain group of organisms, but absent in species outside of this group. The other kind of molecular markers are conserved signature proteins (CSPs) that are specifically present in a monophyletic prokaryotic group. These two molecular markers represent highly reliable characteristics of specific groups of organisms, and they provide novel methods for the identification or delineation of prokaryotic taxonomic units in clear molecular terms (Gao and Gupta, 2012b; Ho et al., 2016; Zhang et al., 2016; Alnajar and Gupta, 2017). We recently identified these molecular markers for *Acidimicrobiia*, another deep-branch class within the phylum *Actinobacteria*, which proved very useful for defining the whole class or different lineages within it and also provide interesting targets for functional studies of these microbes (Hu et al., 2018).

Here, we constructed a phylogenomic tree for 12 sequenced members of the class *Thermoleophilia* based on concatenation of 54 widely distributed conserved proteins. This tree clearly resolved the interrelationship of not yet cultivated species with reconstructed genomes and known type species. More importantly, by analyzing the sequenced *Thermoleophilia* species, we discovered 12 CSIs in different proteins and 32 CSPs that are specific to all members of this class. In addition, a large number of CSIs or CSPs were identified to be unique to certain lineages within this class. This study represents the first and most comprehensive phylogenetic analysis of the class *Thermoleophilia*, and the identified CSIs and CSPs provide valuable molecular markers for the identification and delineation of species belonging to this class or its subordinate taxa.

## Materials and Methods

### Phylogenetic Analysis

A phylogenomic tree for 6 completely sequenced species and 6 metagenome-assembled genomes (MAGs) of the class *Thermoleophilia* (Supplementary Table 1) was constructed. These 6 MAGs were selected for phylogenomic analysis since most single copy orthologous proteins as proposed by Na et al. (2018) can be retrieved from these genomes while other MAGs lack many of these orthologs which will reduce the robustness of the phylogenetic analysis. The deep-branching order *Gaiellales* only has one species sequenced, *Gaiella occulta* F2-233, which was also added to the analyses. The final tree was based on the concatenation of 54 protein sequence alignments (Supplementary Table 2). In addition, sequences from 3 *Rubrobacter* species was used as outgroup to root the tree. Multiple sequence alignments for each protein were performed using the Clustal X 2.1 program (Larkin et al., 2007) and concatenated to produce a single alignment. Gblocks 0.91b program was applied to remove the poorly aligned regions (Talavera and Castresana, 2007) and the resulting alignment composed of 13,132 amino acids was used for phylogenetic analysis. A maximum-likelihood (ML) tree was constructed by MEGA 6.0 with the Whelan and Goldman substitution model based on 1000 bootstrap replicates (Tamura et al., 2013).

An ML tree based on 16S rRNA gene sequences was constructed for the representative strains of *Thermoleophilia* and deep-branching order *Gaiellales*, but no full length 16S rRNA sequences are available for the 6 MAGs. All the 16S rRNA sequences were obtained from Ribosomal Database Project (Cole et al., 2014) or NCBI GenBank, and accession number of each 16S rRNA sequences were summarized in Supplementary Table 3. Sequences from 8 *Rubrobacter* species were used as outgroup to root the tree. The tree was constructed by MEGA 6.0 using the General Time Reversible model with 1000 bootstrap replicates.

### Identification of CSIs

CSIs were identified following the detailed method description by Gupta (Gupta, 2014). Briefly, BLASTP searches were performed on all protein sequences from the genome of *Thermoleophilum album* ATCC 35263 (Yakimov et al., 2003) against all sequences in the NCBI non-redundant protein sequences (nr) database, during the period from January to April, 2018. The general parameters used for BLASTP searches were default as shown in the NCBI website. Multiple sequence alignments were created for homologs of all available *Thermoleophilia* species and a few other bacteria by the Clustal X 2.1 program using default parameters. These sequence alignments were inspected for any conserved insertions or deletions that were restricted to *Thermoleophilia* species only and also flanked by at least 5–6 identical or conserved residues in the neighboring 30∼40 amino acids on each side. The indels with non-conserved flanking regions were not considered. To verify the specificity of the identified indels, another round of BLASTP searches were performed with a short indel-containing fragment (60–100 amino acids long) against the GenBank database. To further confirm that the identified signatures are restricted to *Thermoleophilia* homologs, the top 500 BLAST hits with the highest similarity to the query sequence were inspected for the presence or absence of these CSIs. Final alignment files were generated by two softwares Sig_Create and Sig_Style ^1^ (Gupta, 2014). Due to page limitation, indels-containing sequence alignment in all figures and Supplementary Figures only include those that are found in all *Thermoleophilia* sequences and few sequences from representative strains of other bacteria.

### Identification of CSPs

BLASTP searches were performed on individual proteins from the genome of *T. album* ATCC 35263 to identify proteins that are restricted to species of the class *Thermoleophilia* or the order *Thermoleophilales*. For CSPs that are specific to the order *Solirubrobacterales* or its subgroups at different taxonomic levels, the proteins from the genome of *Patulibacter americanus* DSM 16676 (Reddy and Garcia-Pichel, 2009) were selected as query sequences to do the BLASTP searches against all available sequences in the NCBI non-redundant protein sequences (nr) database. The parameters used for BLASTP searches were generally default except that “Max target sequences” were set to be 500. The BLAST results were manually examined for putative *Thermoleophilia* -specific proteins based on Expected values (*E*-values) (Altschul et al., 1997). Only proteins with significant hits (*E*-values less than 0.01) merely from *Thermoleophilia* genomes while no other hits or hits from non-*Thermoleophilia* genomes generally with *E*-value higher than 1.0 were considered as CSPs in this work (Gao et al., 2006; Gao and Gupta, 2012b).

## Results and Discussion

### Phylogenomic Analysis of the Class *Thermoleophilia*

**FIGURE 1:**
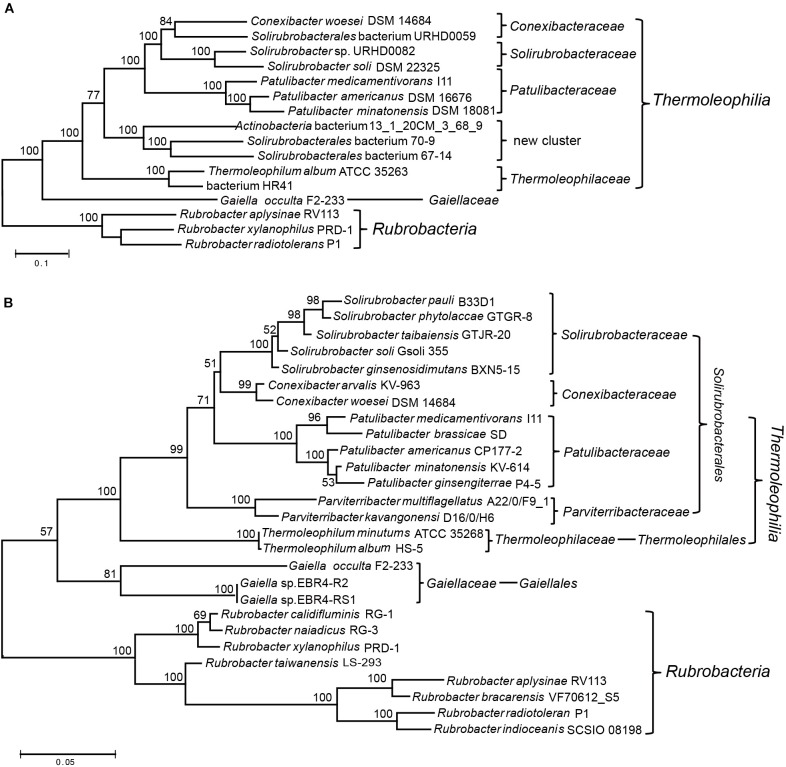
Phylogenetic analysis of the class *Thermoleophilia*. **(A)** Maximum-likelihood tree for *Thermoleophilia* species based upon concatenated sequences of 54 conserved proteins. **(B)** Maximum-likelihood tree based on full length 16S rRNA gene sequences of all type species within the class *Thermoleophilia.* Bootstrap values (%) are shown at each node and different clusters that are consistently observed in both phylogenetic trees are marked.

Two recent comprehensive phylogenetic analyses of the *Actinobacteria* phylum have both applied phylogenomic methods to re-examine the evolutionary relationships or taxonomic framework of species within this phylum (Nouioui et al., 2018; Salam et al., 2019). However, both studies aimed at the entire phylogenetic structure of the phylum, only type species/strains were considered in their analyses. For the poorly represented *Thermoleophilia*, there are only 5∼6 species included in both studies (Nouioui et al., 2018; Salam et al., 2019). Therefore, a comprehensive phylogenomic analysis of the *Thermoleophilia* class is still lacking in spite of the availability of reconstructed genomes for not yet cultivated species of this class. In addition, for these assembled genomes, their exact phylogenetic relationship with type species or taxonomic assignment need to be examined although their association with this class has been suggested (Cabello-Yeves et al., 2018; Woodcroft et al., 2018). Here, we constructed a phylogenetic tree for 6 completely sequenced species and 6 MAGs of this class, for which more single-copy ortholog sequences can be retrieved for a robust phylogenomic analysis (Supplementary Table 1). Finally, 54 orthologous protein sequences that mainly belong to the functional category “translation and transcription” were extracted for the above 12 genomes (Supplementary Table 2) and ML analysis was carried out for the concatenated protein dataset. To our knowledge, this is the most comprehensive phylogenetic analysis for the class *Thermoleophilia* (Figure 1A). In comparison with the current taxonomic framework, we also constructed a phylogenetic tree based on 16S rRNA sequences for this class (Figure 1B). However, surprisingly no complete 16S rRNA sequence were available for the incomplete genome assemblies selected for the above phylogenomic analyses (except that genome assembly of *Solirubrobacter* sp. URHD0082 contained a partial 643 bp fragment of 16S rRNA).

Overall, the combined protein tree showed a very similar branching pattern to the 16S rRNA tree. All species belonging to *Thermoleophilia* formed a robust cluster, separated from the class *Rubrobacteria*. The position of the deep branching order *Gaiellales* is between the boundary of other *Thermoleophilia* orders and the class *Rubrobacteria* in both trees. The single genome-sequenced species *G. occulta* F2-233 clusters with other *Thermoleophilia* orders with a very high bootstrap score 100% in the phylogenomic tree while showing a lower score 57% in the 16S rRNA tree, which is similar to the previous 16S rRNA analyses using the same *G. occulta* strain (Foesel et al., 2016). Within *Thermoleophilia*, species of the two orders *Thermoleophilales* and *Solirubrobacterales* also formed distinctive clusters in the phylogenomic tree, supporting the current order assignment based on 16S rRNA analyses (Reddy and Garcia-Pichel, 2009; Suzuki and Whitman, 2012, 2015). Compared to the diverse soil-source *Solirubrobacterales*, only one cultivable species *T. album* ATCC 35263 from the order *Thermoleophilales* has been genome sequenced (Yakimov et al., 2003). Our phylogenomic tree revealed that MAG “bacterium HR41” clusters together with *T. album*. The genome of HR41 is reconstructed from metagenomic DNA from high-temperature bioreactors, for which the initial samples were collected from an ammonia-rich geothermal groundwater stream in Japan (Kato et al., 2018). In view of their clustering pattern in the phylogenetic tree and common hot spring isolation environment, it is very likely that HR41 represents a species belonging to the family *Thermoleophiliaceae* or the order *Thermoleophilales*.

Notably, 3 MAGs- “Actinobacteria bacterium 13_1_20CM_3_68_9” from grassland (Butterfield et al., 2016), “*Solirubrobacterales* bacterium 67-14” and “*Solirubrobacterales* bacterium 70-9” from bioreactors (Kantor et al., 2015) form a distinct cluster in the phylogenomic tree, more closely related with other *Solirubacteraceae* families than *Thermoleophilales* (Figure 1A). In view of the branching pattern of these 3 MAGs, it is likely that they represent species of a novel family within the order *Solirubrobacterales.* Alternatively, the phylogenetic position of these MAGs is very similar to the two *Parviterribacter* species in the 16S rRNA tree, raising the possibility that they might be members of the *Parviterribacteraceae* family. However, neither the 16S rRNA of the 3 MAGs nor the genome information from the two *Parviterribacter* species is available at the moment, which preclude further analyses. Future new 16S rRNA or genome sequences from closely related species of either the 3 MAGs or the *Parviterribacteraceae* family are needed to define their relationship. In addition, assembled genomes for two monoisolates from the same study of grassland rhizosphere branched differently in our phylogenomic tree. “*Solirubrobacterales* bacterium URHD0059” clusters together with the type species *Conexibacter woesei* DSM 14684 (Pukall et al., 2010), indicating that it might be a new species belonging to the family *Conexibacteraceae*; while “*Solirubrobacter* sp. URHD0082” clusters with *S. soli* DSM 22325 with 100% bootstrap support, demonstrating its affiliation with the family *Solirubacteraceae*. The later association is also confirmed by the 16S rRNA tree based on partial sequence alignment (Supplementary Figure S1). Taken together, these phylogenomic analyses based on a concatenated protein dataset support current taxonomic structure of the class *Thermoleophilia* based on 16S rRNA analyses. In addition, it revealed a new cluster composed of not yet cultivated species that might be a novel family within the order *Solirubrobacterales*.

### Molecular Markers Unique to the Class *Thermoleophilia*

The main purpose of this work is to identify genomic characteristics that are unique to the class *Thermoleophilia* or its subordinate taxa, which can be used to define their taxonomic ranks and also provide targets for functional studies. The complete genome sequences of type species and recently reported MAGs of *Thermoleophilia* are great resources to explore group-specific molecular markers. We focused on two molecular markers as noted earlier: CSIs and CSPs (Gao et al., 2009; Gupta and Gao, 2009; Zhang et al., 2016). Both have been identified for various prokaryotic phyla or other taxonomic ranks higher than genera in the past two decades, and proved to be very useful for phylogenetic and evolutionary studies (Gao and Gupta, 2012b; Ho et al., 2016; Alnajar and Gupta, 2017; Hu et al., 2018).

**FIGURE 2:**
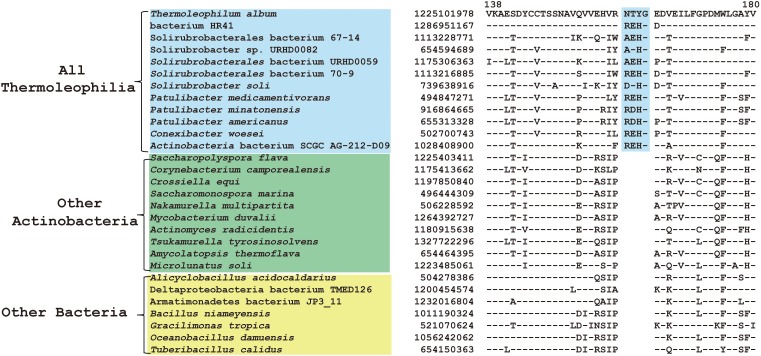
CSI specific to all *Thermoleophilia* species. Partial sequence alignment of the protein quinolinate synthase NadA showing a 4 amino acid insertion in a conserved region that is specific for members of the class *Thermoleophilia*. The dashes in this alignment as well as all other alignments indicate identity with the amino acid on the top line. The GenBank identification numbers of the protein sequences are shown, and the topmost numbers indicate the position of this sequence in the species shown on the top line.

Comparative genomic analyses of species of the class *Thermoleophilia* and other taxonomic units within the kingdom bacteria led to the identification of 12 CSIs in various conserved universal proteins that are only found in *Thermoleophilia* species but not in other bacteria (Table 1). For example, a 4 amino acids (aa) insertion in a very conserved region of quinolinate synthase NadA was specifically shared by *Thermoleophilia* species (Figure 2). NadA is a widely distributed protein in both Archaea and Bacteria and highly conserved due to its important role in nicotinamide adenine dinucleotide (NAD) *de novo* biosynthesis (Ollagnier-De Choudens et al., 2005). A 4aa insertion that is located in a surface loop region of the 3D structure (Volbeda et al., 2016) is only found in homologs from *Thermoleophilia* but not from species outside this class. Therefore, this 4-aa insertion is a distinctive characteristic of the *Thermoleophilia* class. Sequence information for additional 11 CSIs that are specific to all members of this class including assembled genomes of not yet cultivated species is provided in Supplementary Figures S2–S12. In view of their specificity, these CSIs can serve as molecular markers to define and distinguish species belonging to the *Thermoleophilia* class. In addition, none of these 12 CSIs are found in the genome of *Gaiella occulta* F2-233, which is the only genome recently available from the deep-branching order *Gaiellales*.

**Table 1:** Characteristic of Conserved Signature Indels specific to the class *Thermoleophilia* or its associated taxa.

Protein name	GI no.^a^	Figure number	Indel size	Indel position^b^	Specificity
Quinolinate synthase NadA	1225101978	Figure 2	4aa ins^c^	138–180	All *Thermoleophilia*
30S ribosomal protein S10	1093219170	Supplementary Figure S2	1aa ins	72–105	All *Thermoleophilia*
Glutamate-1-semialdehyde-2,1-aminomutase	1225102988	Supplementary Figure S3	2aa del	172–209	All *Thermoleophilia*
D-tyrosyl-tRNA(Tyr) deacylase	1225105696	Supplementary Figure S4	6aa del	100–135	All *Thermoleophilia*
Vitamin B12-dependent ribonucleotide reductase	1225104123	Supplementary Figure S5	1aa ins	746–793	All *Thermoleophilia*
DNA-directed RNA polymerase subunit beta	1225103324	Supplementary Figure S6	2aa ins	215–256	All *Thermoleophilia*
PspA/IM30 family protein	654611971	Supplementary Figure S7	3aa del	184–227	All *Thermoleophilia*
Glutamine-hydrolyzing GMP synthase	1225105599	Supplementary Figure S8	1aa ins	406–450	All *Thermoleophilia*
Elongation factor P	1225104642	Supplementary Figure S9	1aa ins	127–176	All *Thermoleophilia*
Replicative DNA helicase	1225103017	Supplementary Figure S10	2aa ins	15–55	All *Thermoleophilia*
Phenylalanine–tRNA ligase subunit alpha	654610443	Supplementary Figure S11	2–10aa ins	244–285	All *Thermoleophilia*
DNA polymerase III alpha subunit	1225105080	Supplementary Figure S12	1aa ins	84–128	All *Thermoleophilia*
Arginine–tRNA ligase	1225101858	Figure 3	7aa ins	314–367	*Thermoleophiliaceae*
LytR family transcriptional regulator	1225102507	Supplementary Figure S13	2aa ins	155–190	*Thermoleophiliaceae*
DNA gyrase subunit A	1225102941	Supplementary Figure S14	8aa ins	250–298	*Thermoleophiliaceae*
Chaperonin GroEL	1225103134	Supplementary Figure S15	3aa ins	459–497	*Thermoleophiliaceae*
Short chain dehydrogenase	1225103641	Supplementary Figure S16	2aa ins	222–264	*Thermoleophiliaceae*
Type II secretion system F family protein	1225104607	Supplementary Figure S17	1aa ins	299–342	*Thermoleophiliaceae*
Leucyl-tRNA synthetase	1093217654	Supplementary Figure S18	1aa ins	429–469	*Thermoleophiliaceae*
NADH-quinone oxidoreductase subunit B	551309834	Figure 4	1aa del	137–181	*Conexibacteraceae, Solirubrobacteraceae, Patulibacteraceae*
4-hydroxy-3-methylbut-2-enyl diphosphate reductase	739551922	Supplementary Figure S19	1aa ins	44–91	*Conexibacteraceae, Solirubrobacteraceae, Patulibacteraceae*
Pyruvate kinase	652636441	Supplementary Figure S20	5aa del	189–227	*Conexibacteraceae, Solirubrobacteraceae, Patulibacteraceae*
tRNA guanosine (34) transglycosylase Tgt	654594575	Supplementary Figure S21	1aa ins	312–357	*Conexibacteraceae, Solirubrobacteraceae, Patulibacteraceae*
Excinuclease ABC subunit UvrB	654612298	Supplementary Figure S22	1aa ins	215–263	*Conexibacteraceae, Solirubrobacteraceae, Patulibacteraceae*
Transcription antitermination factor NusB	494847549	Supplementary Figure S23	6aa ins	62–102	*Conexibacteraceae, Solirubrobacteraceae, Patulibacteraceae*
Thioredoxin-disulfide reductase	916615184	Figure 5	1aa ins	40–82	*Conexibacteraceae*
Trigger factor	917589205	Supplementary Figure S24	5aa ins	169–217	*Conexibacteraceae*
		Supplementary Figure S25	1aa ins	215–255	*Conexibacteraceae*
Glutamate-5-semialdehyde dehydrogenase	652642436	Supplementary Figure S26	5aa del	150–196	*Conexibacteraceae*
Glutamine amidotransferase	654598081	Figure 6	3aa ins	170–211	*Solirubrobacteraceae*
7,8-didemethyl-8-hydroxy-5-deazariboflavin synthase subunit CofH	654594367	Supplementary Figure S27	4aa del	152–192	*Solirubrobacteraceae*
methionine–tRNA ligase	654600348	Supplementary Figure S28	5aa ins	267–310	*Solirubrobacteraceae*
Asp-tRNA(Asn)/Glu-tRNA(Gln) amidotransferase subunit GatC	654597239	Supplementary Figure S29	1aa ins	20–65	*Solirubrobacteraceae*
CTP synthase	921290543	Supplementary Figure S30	2aa ins	264–308	*Solirubrobacteraceae*
DNA-directed RNA polymerase subunit beta’	494853285	Figure 7	8aa ins	376–420	*Patulibacteraceae*
SDR family NAD(P)-dependent oxidoreductase	494848053	Supplementary Figure S31	2aa ins	149–198	*Patulibacteraceae*
Dihydrolipoyl dehydrogenase	551307243	Supplementary Figure S32	1aa del	355–396	*Patulibacteraceae*
Methylmalonyl-CoA epimerase	551310266	Supplementary Figure S33	2aa ins	1–48	*Patulibacteraceae*
Acetyl-CoA carboxylase biotin carboxylase subunit	551309981	Supplementary Figure S34	2aa ins	224–268	*Patulibacteraceae*
GTPase HflX	1225104795	Supplementary Figure S35	1aa ins	282–322	*Patulibacteraceae*
1-deoxy-D-xylulose-5-phosphate reductoisomerase	551310630	Supplementary Figure S36	6–8aa ins	146–188	*Patulibacteraceae*
Tryptophan–tRNA ligase	494851195	Supplementary Figure S37	4–12aa ins	152–191	*Patulibacteraceae*
Endopeptidase La	551309049	Supplementary Figure S38	1aa ins	228–266	*Patulibacteraceae*
7,8-didemethyl-8-hydroxy-5-deazariboflavin synthase subunit CofH	494847285	Supplementary Figure S39	4aa ins	481–522	*Patulibacteraceae*
NADH-quinone oxidoreductase subunit I	1113228917	Supplementary Figure S40	1aa ins	72–125	New cluster
Adenylosuccinate synthase	1113229450	Supplementary Figure S41	17–23aa ins	154–204	S.67-14 and S.70-9^d^
GTPase Era	1113226493	Supplementary Figure S42	1–2aa ins	38–88	S.67-14 and S.70-9
Heme-copper oxidase subunit III	1113215223	Supplementary Figure S43	1–4aa ins	121–167	S.67-14 and S.70-9

*^a^The GI number represents the GenBank identification number of the protein sequence from one Thermoleophilia species that contain the specific CSI. ^b^The indel region indicates the region of the protein where the described CSI is present. ^c^ins, insertion; del, deletion. ^d^S.67-14 and S.70-9 are abbreviations for MAG “Solirubrobacterales bacterium 67-14” and “Solirubrobacterales bacterium 70-9.”*

Except the CSIs, we performed BLASTp searches for each protein from the type species *T. album* ATCC 35263 to identify CSPs that are specific to the *Thermoleophilia* class. In total, 32 proteins are uniquely shared by almost all sequenced *Thermoleophilia* genomes but not found in any other bacterial taxa except 4 present in *G. occulta* F2-233 (Table 2). Foesel et al. have proposed that *Gaiellales* is a deeply branching order within the class *Thermoleophilia* based on 16S rRNA analyses and some shared phenotypic features of one single strain *G. occulta* F2-233 and other *Thermoleophilia/Rubrobacteria* species (Foesel et al., 2016). The presence of 4 CSPs in the same *G. occulta* strain could be derived from the common ancestor of *Gaiellales* with the other *Thermoleophilia* orders or due to lateral gene transfer, which awaits confirmation from more genomes of the *Gaiellales*. Additionally, 3 proteins are missing in the MAGs from the newly defined potential family based on our phylogenomic analysis presented in Figure 1A but found in the other members of the class, which is possibly due to incomplete genome information. Indeed, the assembly qualities of MAGs varies as indicated by the summary of Contig-N50 statistic values in Supplementary Table 1. Therefore, it is very likely that the identified 3 CSPs are present in the species of the newly defined cluster, while the MAGs did not cover the sequence region. Together with the identified CSIs, these CSPs are additional molecular markers for *Thermoleophilia.* It should be mentioned that all these identified CSPs are hypothetical proteins with unknown function. Since they are restricted to species of *Thermoleophilia*, functional studies on them may uncover biochemical or physiological features that are unique to this class.

**Table 2:** Conserved Signature Proteins that are uniquely found in the *Thermoleophilia* class.

Protein product	Length	Specificity	Function
**(A) CSPs uniquely present in All *Thermoleophilia* species (29)^a^**			
WP_093115104.1	242	All *thermoleophilia*	Unknown
WP_093115134.1	90	*All thermoleophilia*	Unknown
WP_093115673.1	127	All *thermoleophilia*	Unknown
WP_093115681.1	103	All *thermoleophilia*	Unknown
WP_093115745.1	166	All *thermoleophilia*	Unknown
WP_093115827.1	993	All *thermoleophilia*	Unknown
WP_093116216.1	151	All *thermoleophilia*	Unknown
WP_093116230.1	213	All *thermoleophilia*	Unknown
WP_093116634.1	159	All *thermoleophilia*	Unknown
WP_093116636.1^b^	64	All *thermoleophilia*	Unknown
WP_093116642.1	114	All *thermoleophilia*	Unknown
WP_093116769.1	130	All *thermoleophilia*	Unknown
WP_093116819.1	167	All *thermoleophilia*	Unknown
WP_093116917.1	120	All *thermoleophilia*	Unknown
WP_093116997.1	185	All *thermoleophilia*	Unknown
WP_093117023.1	151	All *thermoleophilia*	Unknown
WP_093117047.1	572	All *thermoleophilia*	Unknown
WP_093117060.1	247	All *thermoleophilia*	Unknown
WP_093117260.1	72	All *thermoleophilia*	Unknown
WP_093117458.1^b^	142	All *thermoleophilia*	Unknown
WP_093117523.1	269	All *thermoleophilia*	Unknown
WP_093118104.1	79	All *thermoleophilia*	Unknown
WP_093118304.1^b^	132	All *thermoleophilia*	Unknown
WP_093118364.1^b^	257	All *thermoleophilia*	Unknown
WP_093118537.1	154	All *thermoleophilia*	Unknown
WP_093118589.1	178	All *thermoleophilia*	Unknown
WP_093118635.1	120	All *thermoleophilia*	Unknown
WP_093118833.1	82	All *thermoleophilia*	Unknown
WP_093119001.1	187	All *thermoleophilia*	Unknown
**(B) CSPs unique to *Thermoleophilia* class but not found in new cluster (3)**			
WP_093116803.1	141	*Thermoleophilia* except new cluster	Unknown
WP_093118036.1	211	*Thermoleophilia* except new cluster	Unknown
WP_093116745.1	226	*Thermoleophilia* except new cluster	Unknown

*^a^The number in brackets represents the total number of CSPs unique to the specific group. ^b^Four CSPs are also present in the genome of Gaiella occulta F2-233 (GenBank accession: GCA_003351045.1).*

### Molecular Signatures for Major Lineages Within *Thermoleophilia*

**FIGURE 3:**
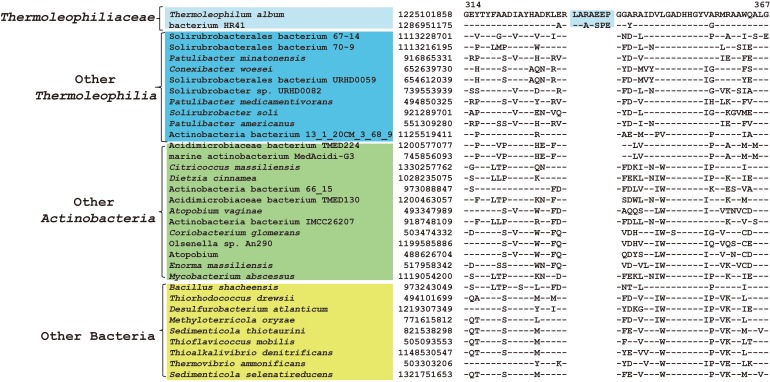
CSI specific to *T. album* and MAG HR41. Partial sequence alignment of arginine–tRNA ligase showing a 7 amino acid insertion that is uniquely shared by *T. album* and MAG HR41.

As described earlier, the order *Thermoleophilales* or its sole family *Thermoleophiliaceae* only have two genomes available, including *T. album* ATCC 35263 and MAG “bacterium HR41.” Our analyses identified 7 CSIs in different proteins (Table 1) and 29 CSPs (Table 3) that are only present in these two genomes but absent in other bacteria. Figure 3 shows one example of these CSIs. In the sequence alignment of arginine-tRNA ligase, a 7aa insertion flanked by highly conserved residues is uniquely found in homologs from both *T. album* and MAG “bacterium HR41.” Sequence information for further 6 CSIs with the same specificity are shown in Supplementary Figures S13–S18. Whether these identified CSIs and CSPs can constitute distinctive markers for the *Thermoleophiliaceae* family or even the *Thermoleophilales* order awaits confirmation from more sequences of other species belonging to this lineage. Nevertheless, these results provide additional evidence for the close relationship of MAG “bacterium HR41” and *T. album*.

**Table 3:** Conserved Signature Proteins that are uniquely found in the subgroups of *Thermoleophilia* class.

Accession no.	Length	Specificity
**(A) CSPs uniquely present in family *Thermoleophilaceae* (29)^a^**		
WP_093115090.1	197	*Thermoleophilaceae*
WP_093115144.1	179	*Thermoleophilaceae*
WP_093115294.1	164	*Thermoleophilaceae*
WP_093115296.1	180	*Thermoleophilaceae*
WP_093115479.1	319	*Thermoleophilaceae*
WP_093115661.1	93	*Thermoleophilaceae*
WP_093115901.1	156	*Thermoleophilaceae*
WP_093115943.1	202	*Thermoleophilaceae*
WP_093116532.1	154	*Thermoleophilaceae*
WP_093116727.1	429	*Thermoleophilaceae*
WP_093116780.1	110	*Thermoleophilaceae*
WP_093116825.1	68	*Thermoleophilaceae*
WP_093116919.1	83	*Thermoleophilaceae*
WP_093117092.1	264	*Thermoleophilaceae*
WP_093117483.1	93	*Thermoleophilaceae*
WP_093117587.1	83	*Thermoleophilaceae*
WP_093117642.1	114	*Thermoleophilaceae*
WP_093117817.1	157	*Thermoleophilaceae*
WP_093117827.1	199	*Thermoleophilaceae*
WP_093117877.1	136	*Thermoleophilaceae*
WP_093118281.1	403	*Thermoleophilaceae*
WP_093118340.1	146	*Thermoleophilaceae*
WP_093118436.1	119	*Thermoleophilaceae*
WP_093118524.1	170	*Thermoleophilaceae*
WP_093118569.1	80	*Thermoleophilaceae*
WP_093118679.1	148	*Thermoleophilaceae*
WP_093118731.1	93	*Thermoleophilaceae*
WP_093118750.1	573	*Thermoleophilaceae*
WP_093118752.1	195	*Thermoleophilaceae*
**(B) CSPs uniquely present in Conexibacteraceae, Solirubrobacteraceae, and Patulibacteraceae (24)**		
WP_022926981.1	246	*Conexibacteraceae, Solirubrobacteraceae, Patulibacteraceae*
WP_022926986.1	115	*Conexibacteraceae, Solirubrobacteraceae, Patulibacteraceae*
WP_022927172.1	216	*Conexibacteraceae, Solirubrobacteraceae, Patulibacteraceae*
WP_022927347.1	417	*Conexibacteraceae, Solirubrobacteraceae, Patulibacteraceae*
WP_022927380.1	114	*Conexibacteraceae, Solirubrobacteraceae, Patulibacteraceae*
WP_022927389.1	468	*Conexibacteraceae, Solirubrobacteraceae, Patulibacteraceae*
WP_022927525.1	461	*Conexibacteraceae, Solirubrobacteraceae, Patulibacteraceae*
WP_022927538.1	181	*Conexibacteraceae, Solirubrobacteraceae, Patulibacteraceae*
WP_022927665.1	153	*Conexibacteraceae, Solirubrobacteraceae, Patulibacteraceae*
WP_022927703.1	253	*Conexibacteraceae, Solirubrobacteraceae, Patulibacteraceae*
WP_022927703.1	253	*Conexibacteraceae, Solirubrobacteraceae, Patulibacteraceae*
WP_022927792.1	224	*Conexibacteraceae, Solirubrobacteraceae, Patulibacteraceae*
WP_022927799.1	564	*Conexibacteraceae, Solirubrobacteraceae, Patulibacteraceae*
WP_022927801.1	265	*Conexibacteraceae, Solirubrobacteraceae, Patulibacteraceae*
WP_022928134.1	160	*Conexibacteraceae, Solirubrobacteraceae, Patulibacteraceae*
WP_022928438.1	136	*Conexibacteraceae, Solirubrobacteraceae, Patulibacteraceae*
WP_022928438.1	136	*Conexibacteraceae, Solirubrobacteraceae, Patulibacteraceae*
WP_022929183.1	133	*Conexibacteraceae, Solirubrobacteraceae, Patulibacteraceae*
WP_022929536.1	104	*Conexibacteraceae, Solirubrobacteraceae, Patulibacteraceae*
WP_022929558.1	227	*Conexibacteraceae, Solirubrobacteraceae, Patulibacteraceae*
WP_022930026.1	369	*Conexibacteraceae, Solirubrobacteraceae, Patulibacteraceae*
WP_022930484.1	604	*Conexibacteraceae, Solirubrobacteraceae, Patulibacteraceae*
WP_028721853.1	100	*Conexibacteraceae, Solirubrobacteraceae, Patulibacteraceae*
WP_051160538.1	289	*Conexibacteraceae, Solirubrobacteraceae, Patulibacteraceae*
**(C) CSPs uniquely present in family *Patulibacteraceae* (31)**		
WP_022926969.1	211	*Patulibacteraceae*
WP_022926970.1	304	*Patulibacteraceae*
WP_022927005.1	421	*Patulibacteraceae*
WP_022927132.1	338	*Patulibacteraceae*
WP_022927548.1	105	*Patulibacteraceae*
WP_022927557.1	100	*Patulibacteraceae*
WP_022927572.1	162	*Patulibacteraceae*
WP_022928009.1	773	*Patulibacteraceae*
WP_022928045.1	170	*Patulibacteraceae*
WP_022928129.1	165	*Patulibacteraceae*
WP_022928139.1	176	*Patulibacteraceae*
WP_022928142.1	174	*Patulibacteraceae*
WP_022928143.1	155	*Patulibacteraceae*
WP_022928333.1	248	*Patulibacteraceae*
WP_022928557.1	67	*Patulibacteraceae*
WP_022928588.1	110	*Patulibacteraceae*
WP_022928655.1	62	*Patulibacteraceae*
WP_022928967.1	242	*Patulibacteraceae*
WP_022929153.1	236	*Patulibacteraceae*
WP_022929154.1	209	*Patulibacteraceae*
WP_022929593.1	417	*Patulibacteraceae*
WP_022929618.1	66	*Patulibacteraceae*
WP_022929735.1	411	*Patulibacteraceae*
WP_022929823.1	153	*Patulibacteraceae*
WP_022929914.1	269	*Patulibacteraceae*
WP_022929990.1	171	*Patulibacteraceae*
WP_022930081.1	281	*Patulibacteraceae*
WP_022930294.1	190	*Patulibacteraceae*
WP_022930374.1	124	*Patulibacteraceae*
WP_022930538.1	472	*Patulibacteraceae*
WP_022930714.1	206	*Patulibacteraceae*

*^a^The number in brackets represents the total number of CSPs unique to the specific group.*

**FIGURE 4:**
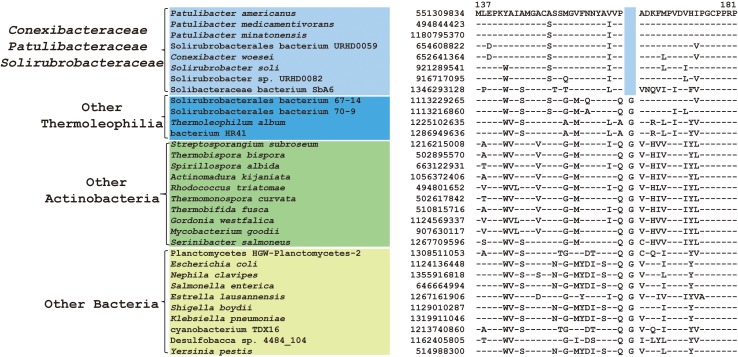
CSI specific to the families *Conexibacteraceae, Solirubrobacteraceae* and *Patulibacteraceae*. Partial alignment of the protein NADH-quinone oxidoreductase subunit B showing a 1 amino acid deletion that is uniquely shared by 3 families *Conexibacteraceae, Solirubrobacteraceae* and *Patulibacteraceae.*

Within the order *Solirubrobacterales*, we have identified 6 CSIs that are specific to species of 3 families including *Conexibacteraceae*, *Solirubacteraceae*, and *Patulibacteraceae*, but no CSIs also shared by members of the new cluster (Table 1). One of these CSIs is illustrated in Figure 4, which is 1 aa deletion in a very conserved fragment of NADH-quinone oxidoreductase subunit B. Sequence information for other 5 CSIs that are uniquely shared by these 3 families are presented in Supplementary Figures S19–S23. Additionally, we discovered 24 CSPs that are only found in genomes of the named above 3 families but not in any other bacteria (Table 3). The shared presence of 6 CSIs and a number of CSPs indicate that *Conexibacteraceae*, *Solirubacteraceae*, and *Patulibacteraceae* are monophyletic. These two kinds of signature sequences were most likely introduced in the common ancestor of these three families and later on passed to all decedents. Moreover, if genome sequence of the fourth family *Parviterribacteraceae* becomes available in the future, it is worthwhile to examine whether some of these CSIs and CSPs are also shared by *Parviterribacteraceae* and actually constitute molecular markers of the *Solirubrobacterales* order.

**FIGURE 5:**
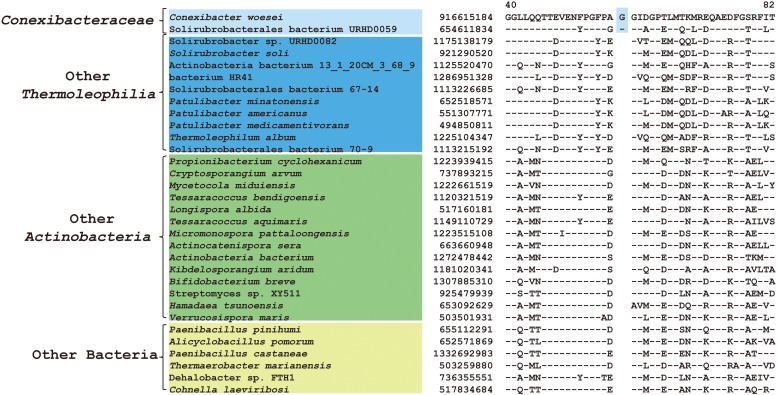
CSI specific to *Conexibacteraceae.* A 1 amino acid insertion in the protein thioredoxin-disulfide reductase that is uniquely shared by *C. woesei* and associated MAG.

**FIGURE 6:**
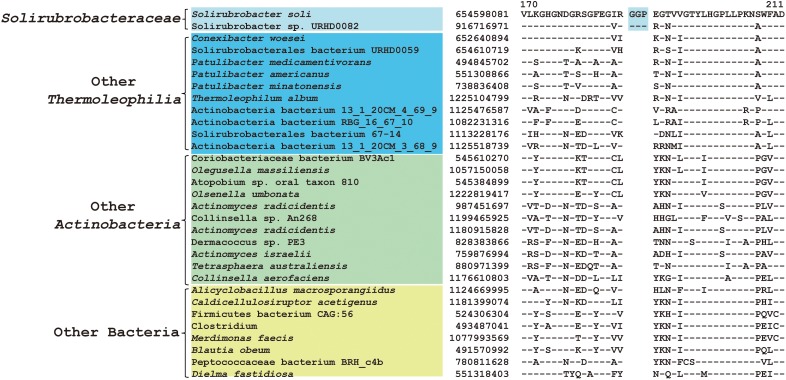
CSI specific to *Solirubrobacteraceae*. A 3 amino acid CSI in the protein glutamine amidotransferase that is specific for *S. soli* and associated MAG.

**FIGURE 7:**
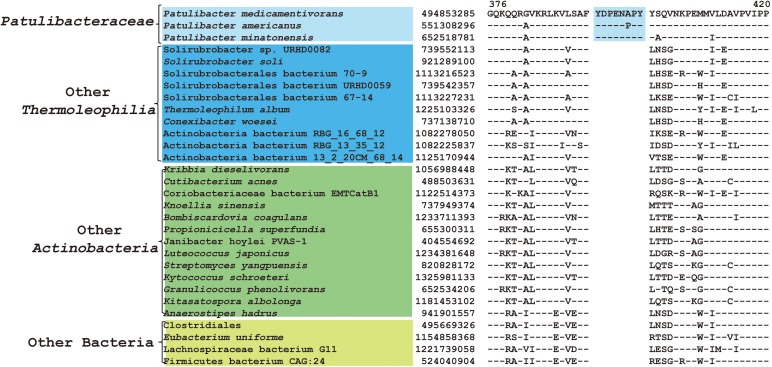
CSI specific to *Patulibacteraceae*. Partial sequence alignment of DNA-directed RNA polymerase subunit beta’ showing an 8 amino acid insertion that is specific for *Patulibacteraceae*.

As mentioned earlier, at family level within *Thermoleophilia*, only few cultivable strains are available and our current descriptions of some families such as *Conexibacteraceae* or *Solirubacteraceae* are only based on 1 or 2 strains. Here, we identified a number of CSIs that are specific to all genome-sequenced members of each family of *Thermoleophilia* except *Parviterribacteraceae* that don’t have genome sequence available (Table 1). For example, 4 CSIs were found to be unique to members of *Conexibacteraceae* (Figure 5 and Supplementary Figures S24–S26), 5 CSIs for *Solirubacteraceae* (Figure 6 and Supplementary Figures S27–S30), and totally 10 CSIs shared by 3 species of *Patulibacteraceae* (Figure 7 and Supplementary Figures S31–S39). We attempted to search for CSIs that are specific to the new cluster revealed by our phylogenomic analysis. Due to the incompleteness of the 3 genome assemblies, only 1 CSI is specifically shared by all three members of the new cluster (Supplementary Figure S40), while another 3 CSIs are only found in MAG “*Solirubrobacterales* bacterium 67-14” and “*Solirubrobacterales* bacterium 70-9” with two protein homologs missing in “Actinobacteria bacterium 13_1_20CM_3_68_9” (Supplementary Figures S41–S43). Furthermore, since more genomes are sequenced for *Patulibacteraceae*, we also identified 31 CSPs that are restricted to the genomes of this family, which provide additional markers for them (Table 3).

## Conclusion

Although metagenomic studies suggest that species of the class *Thermoleophilia* are abundant in hot spring and soil samples and they play an important role in biogeochemical cycling, very few studies have been performed on the phylogeny of this deep branch of *Actinobacteria*. Our current understanding of their taxonomy and phylogeny based on few cultivated species needs to be updated to better serve our exploration of this class. In this work, we have carried out detailed phylogenomic analysis of sequenced *Thermoleophilia* species and assembled genomes. The constructed phylogenetic tree clearly demonstrated the close affiliation of not yet cultivated MAGs with culturable type species. A new robust cluster composed of not yet cultivated MAGs is revealed within this class that might be a novel family belonging to *Solirubrobacterales*. Moreover, we identified a large number of CSIs and CSPs that are either specific to all species of this class or various lineages within it. These two types of signature sequences provide novel molecular markers that can be applied to define or distinguish the class *Thermoleophilia* or its affiliated taxa at higher taxonomic ranks, in addition to the 16S rRNA gene alone based standard.

In addition to their phylogenetic implications, these lineage-specific CSIs and CSPs can also be used to test the presence of *Thermoleophilia* species in different environmental samples. PCR primers could be designed for gene fragments that contain the above described CSIs or genes for CSPs, then we can detect the existence of certain lineages based on the presence or absence of the CSIs and CSPs. Furthermore, the functional significance of all CSIs and CSPs identified from this work are unknown. Due to their specificity to the *Thermoleophilia* class, functional studies on them might lead to identification of biochemical or physiological characteristics that are unique to this class of bacteria.

## Author Contributions

DH carried out comparative analyses of the *Thermoleophilia* genomes to identify signatures reported here and constructed the phylogenetic trees. BG, DH, YZ and YM were responsible for the writing and editing of the manuscript. All of the work was carried out under the direction of BG.

## Conflict of Interest Statement

The authors declare that the research was conducted in the absence of any commercial or financial relationships that could be construed as a potential conflict of interest.

## Footnotes

1 Gleans.net
