# Supplementary material for: Identification of Molecular Markers That Are Specific to the Class Thermoleophilia
Source: Front Microbiol. 2019 May 24;10:1185. doi: 10.3389/fmicb.2019.01185 (PMC6544083; doi:10.3389/fmicb.2019.01185)
Supplement: Supplementary file 1 [file Data_Sheet_1.PDF]

*Supplementary Material*

# Identification of molecular markers that are specific to the class *Thermoleophilia*

**Danyu Hu*, Yang Zang*, Yingjin Mao and Beile Gao#**

*: These authors have contributed equally to the work.

#: Correspondence: gaob@scsio.ac.cn

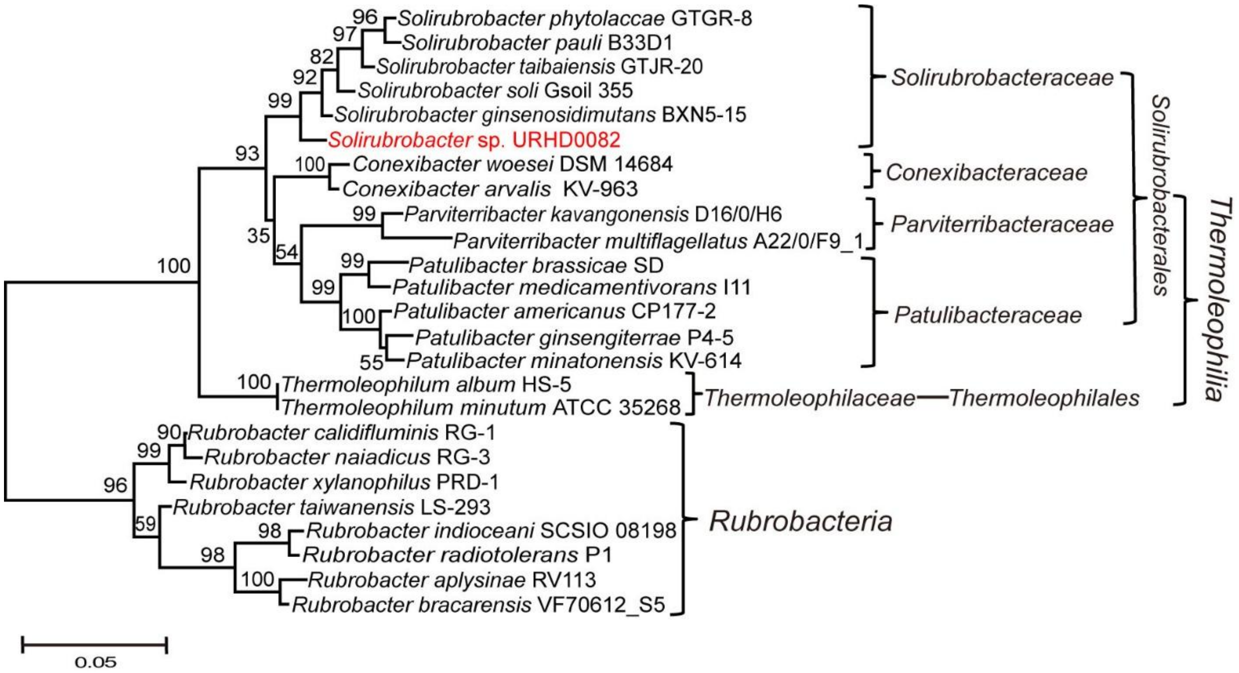


**Supplementary Figure S1.** Maximum-likelihood tree based on partial 16S rRNA gene sequence alignments of all type species and an MAG *Solirubrobacter* sp. URHD0082 within the class *Thermoleophilia*. Bootstrap values (%) are shown at each node.

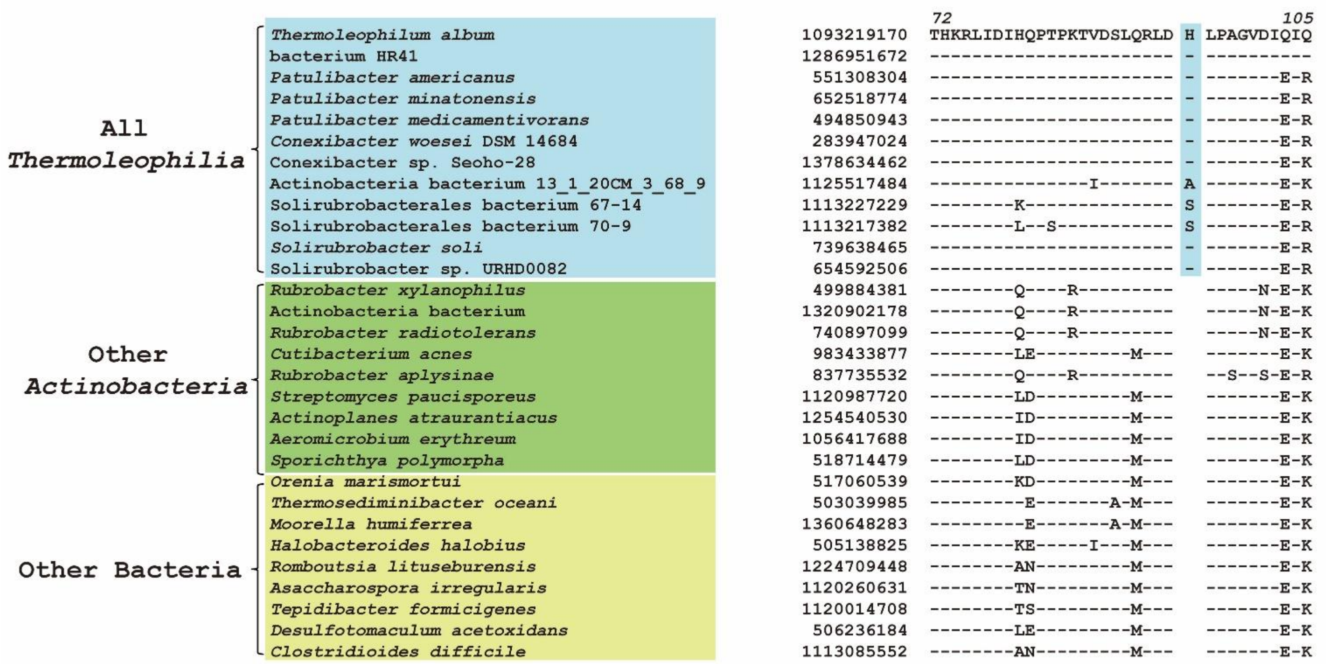


**Supplementary Figure S2.** CSI specific to all *Thermoleophilia* species. Partial sequence alignment of the protein 30S ribosomal protein S10 showing a 1aa insertion in a conserved region that is specific to members of the class *Thermoleophilia*.

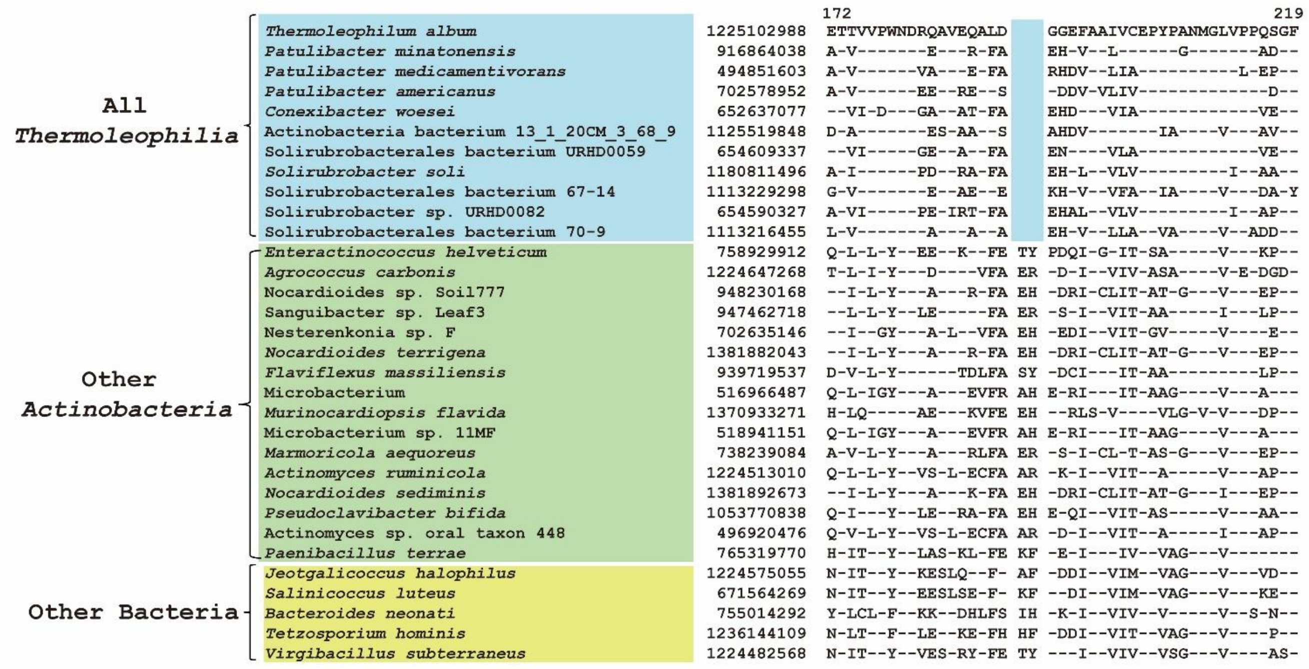


**Supplementary Figure S3.** CSI specific to all *Thermoleophilia* species. Partial sequence alignment of the protein glutamate-1-semialdehyde-2, 1-aminomutase showing a 2aa deletion in a conserved region that is specific to members of the class *Thermoleophilia*.

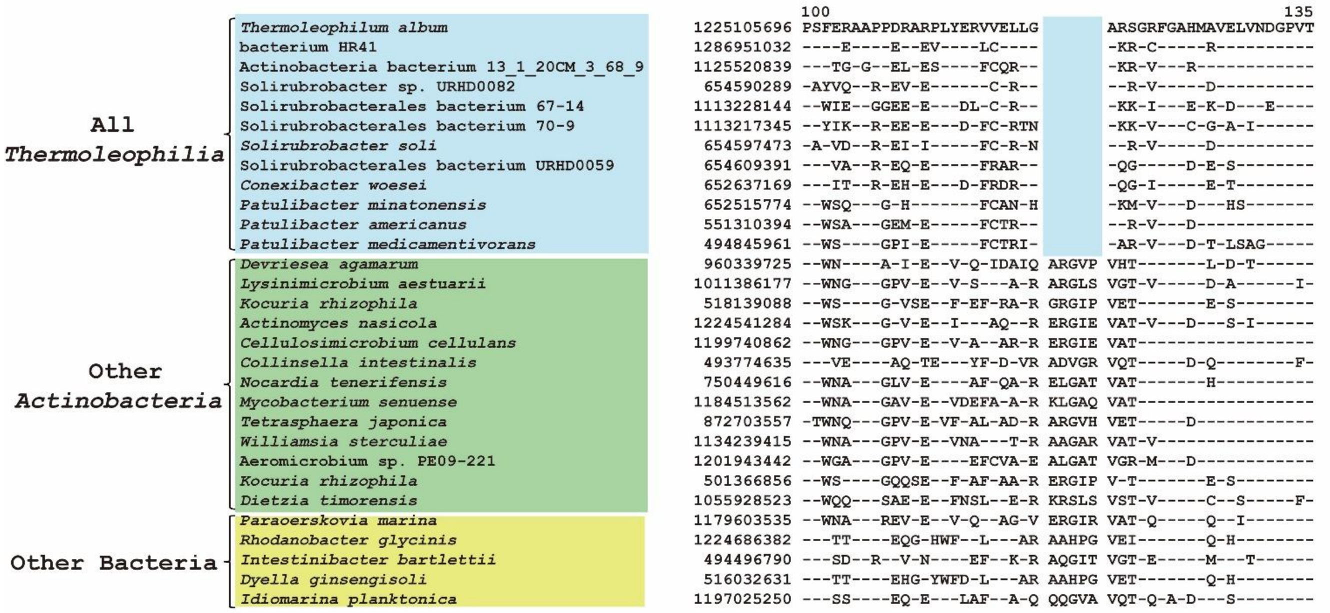


**Supplementary Figure S4.** CSI specific to all *Thermoleophilia* species. Partial sequence alignment of the protein D-tyrosyl-tRNA (Dtyr) deacylase showing a 6aa deletion in a conserved region that is specific to members of the class *Thermoleophilia*.

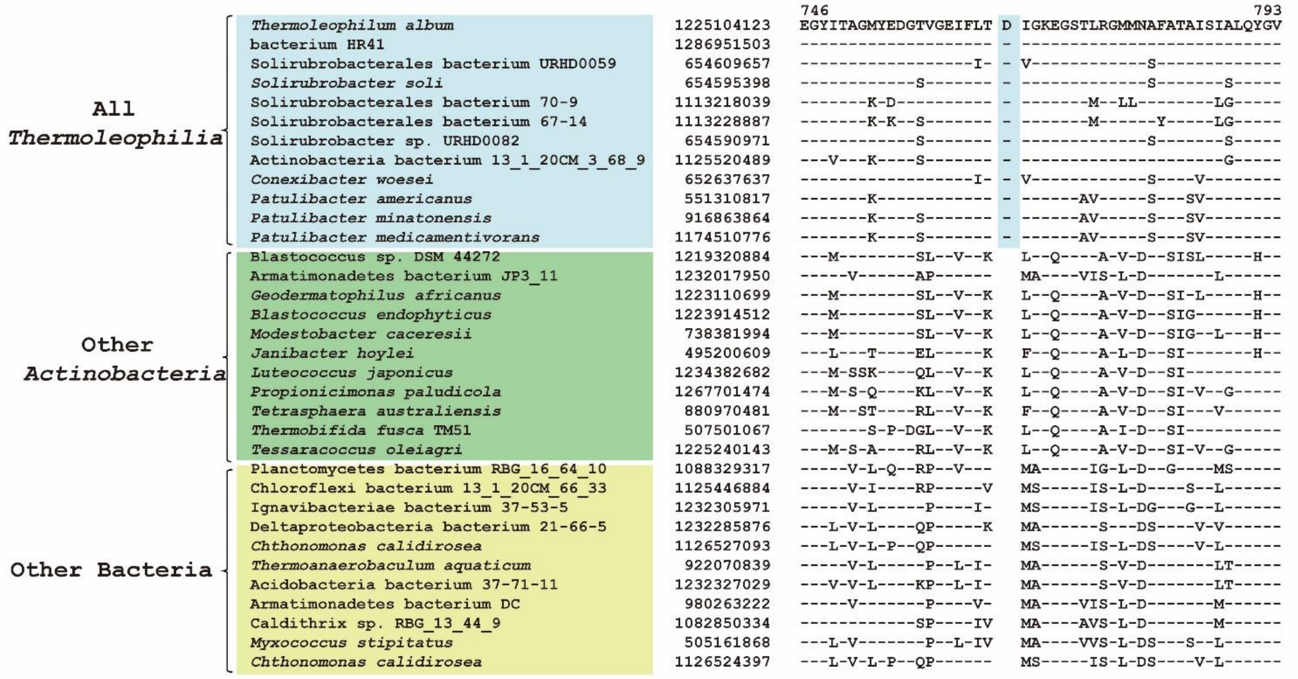


**Supplementary Figure S5.** CSI specific to all *Thermoleophilia* species. Partial sequence alignment of the protein vitamin B12-dependent ribonucleotide reductase showing a 1aa insertion in a conserved region that is specific to members of the class *Thermoleophilia.*

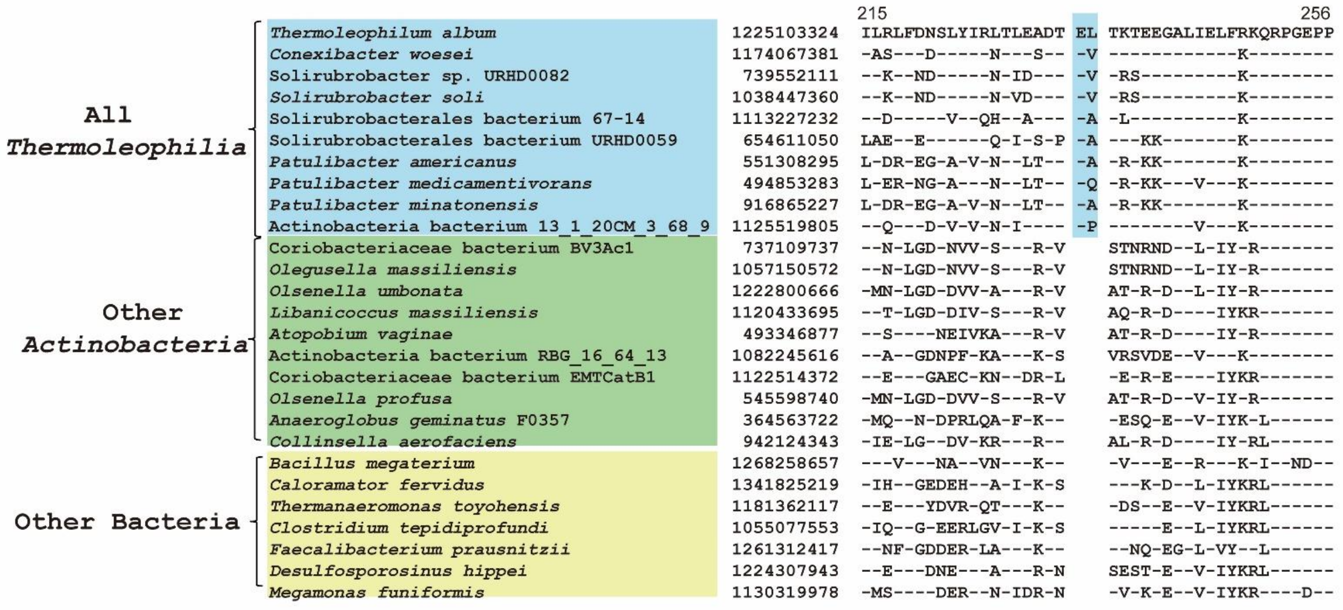


**Supplementary Figure S6.** CSI specific to all *Thermoleophilia* species. Partial sequence alignment of the protein DNA-directed RNA polymerase subunit beta showing a 2aa insertion in a conserved region that is specific to members of the class *Thermoleophilia.*

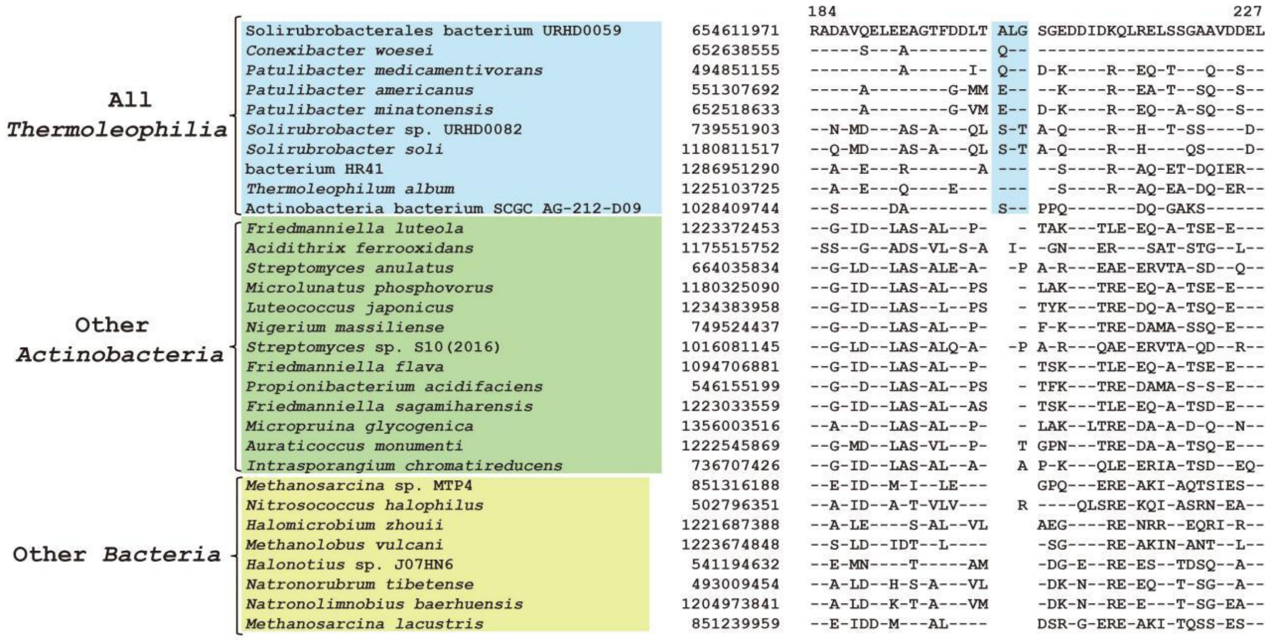


**Supplementary Figure S7.** CSI specific to all *Thermoleophilia* species. Partial sequence alignment of the protein PspA/IM30 family protein showing a 3aa insertion in a conserved region that is specific to members of the class *Thermoleophilia*.

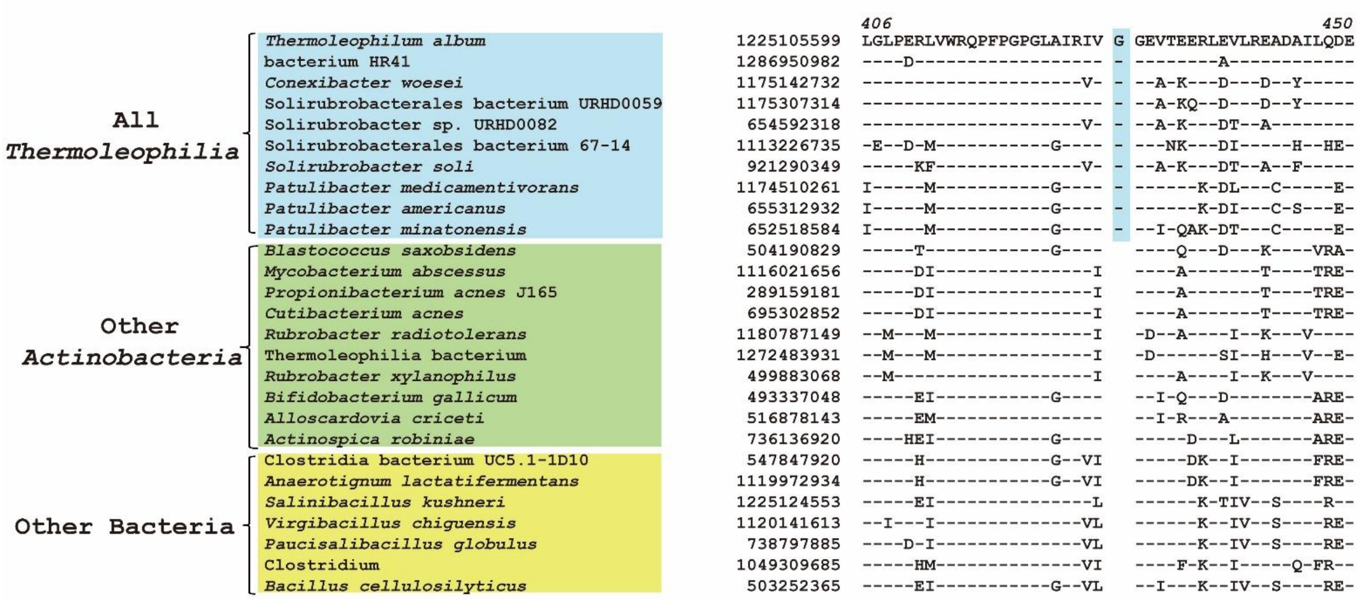


**Supplementary Figure S8.** CSI specific to all *Thermoleophilia* species. Partial sequence alignment of the protein glutamine-hydrolyzing GMP synthase showing a 1aa insertion in a conserved region that is specific to members of the class *Thermoleophilia*.

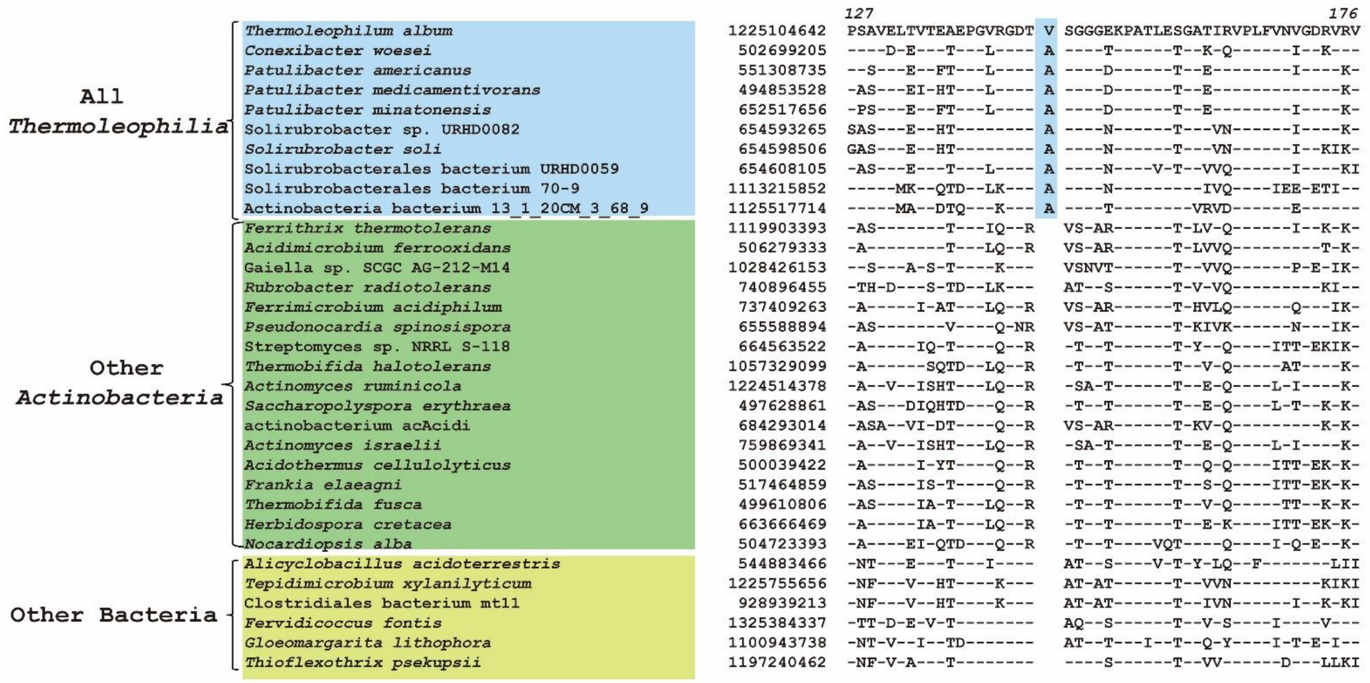


**Supplementary Figure S9.** CSI specific to all *Thermoleophilia* species. Partial sequence alignment of the protein elongation factor P showing a 1aa insertion in a conserved region that is specific to members of the class *Thermoleophilia*.

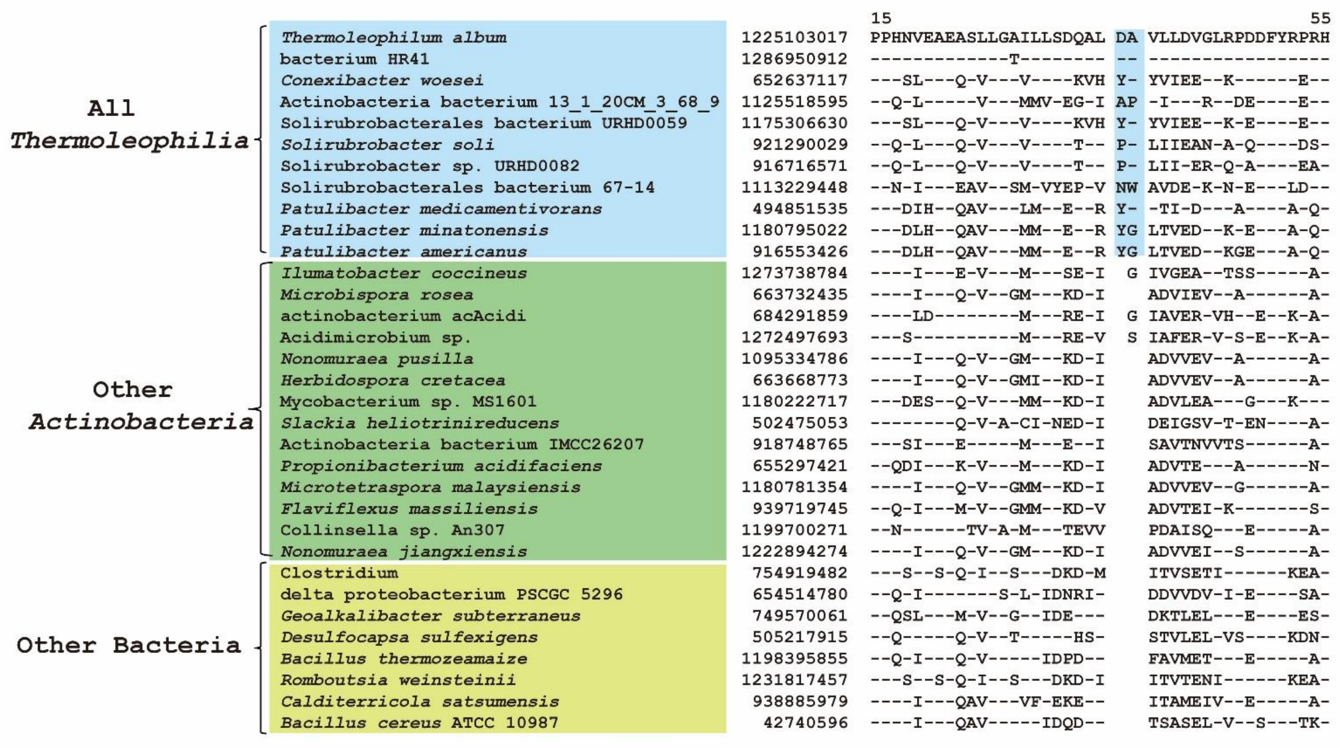


**Supplementary Figure S10.** CSI specific to all *Thermoleophilia* species. Partial sequence alignment of the protein replicative DNA helicase showing a 1-2aa insertion in a conserved region that is specific to members of the class *Thermoleophilia*.

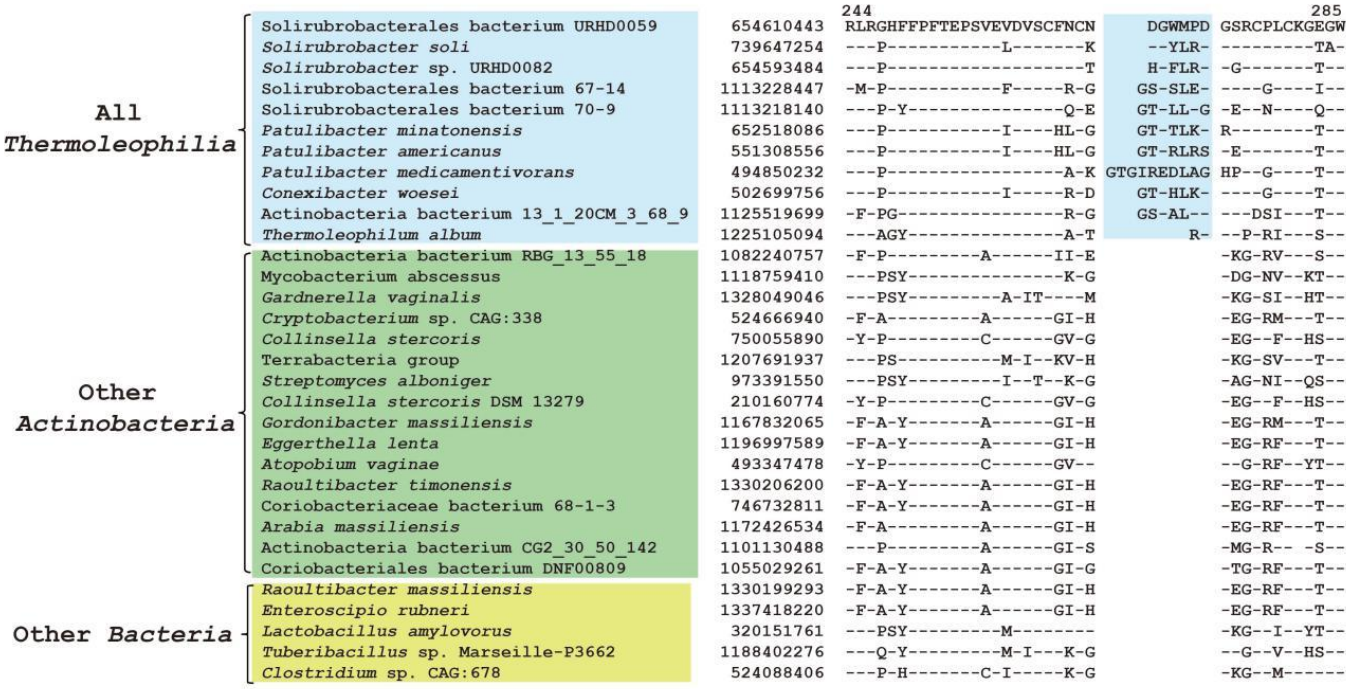


**Supplementary Figure S11.** CSI specific to all *Thermoleophilia* species. Partial sequence alignment of the protein phenylalanine--tRNA ligase subunit alpha showing a 2-10aa insertion in a conserved region that is specific to members of the class *Thermoleophil*

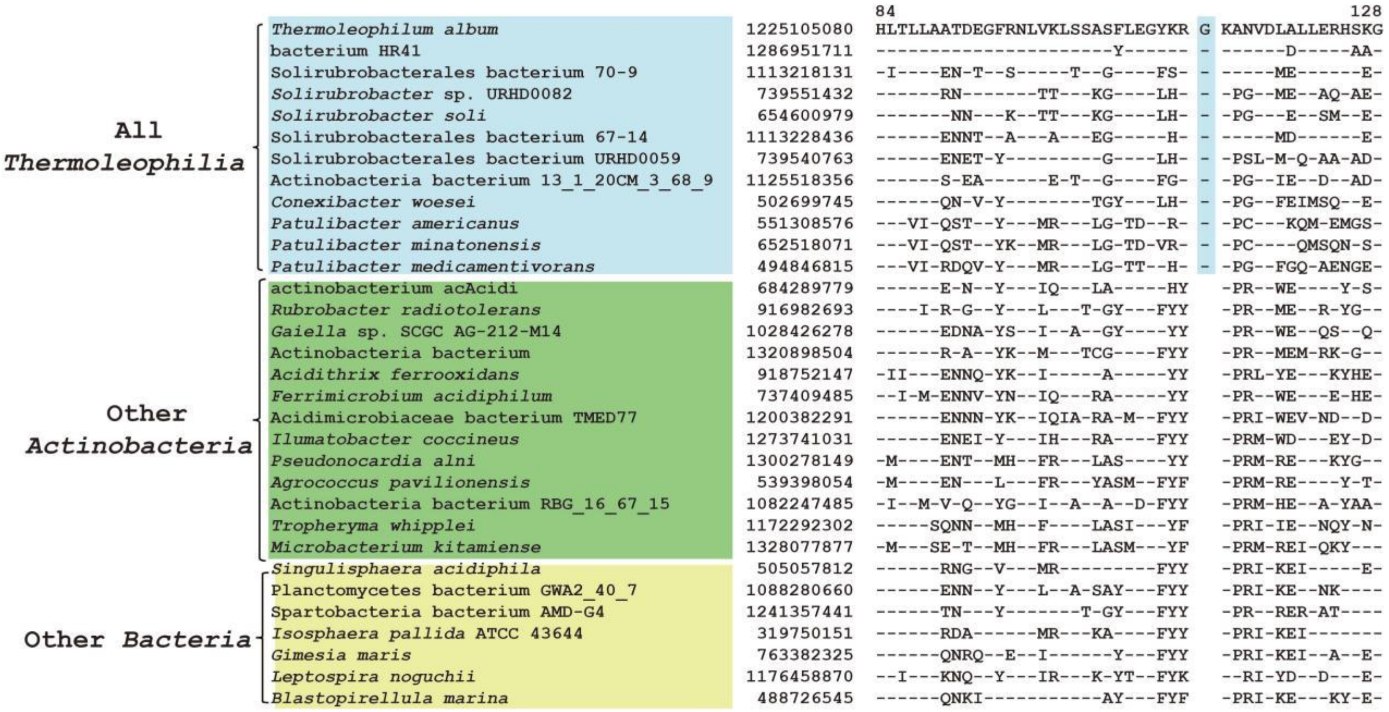


**Supplementary Figure S12.** CSI specific to all *Thermoleophilia* species. Partial sequence alignment of the protein DNA polymerase III alpha subunit showing a 1aa insertion in a conserved region that is specific to members of the class *Thermoleophilia*

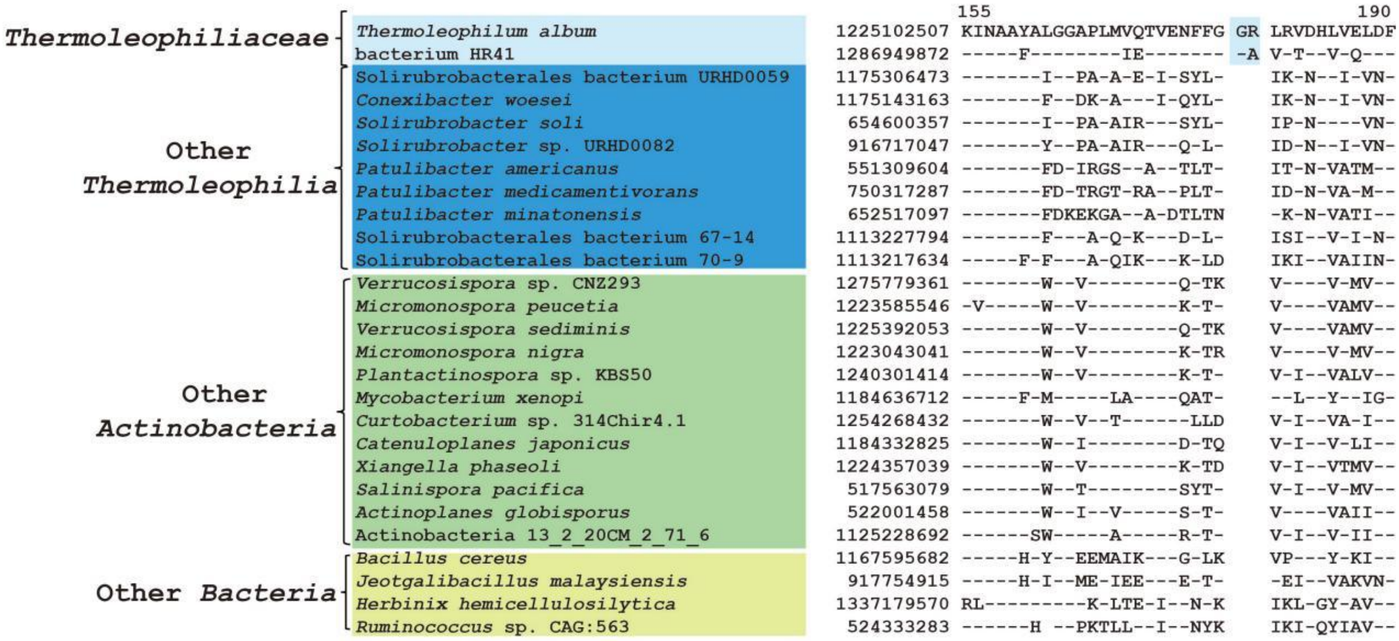


**Supplementary Figure S13.** *Thermoleophilaceae* family specific CSI. Partial alignment of the protein LytR family transcriptional regulator showing a 2aa insertion that is specific to the family *Thermoleophilaceae*.

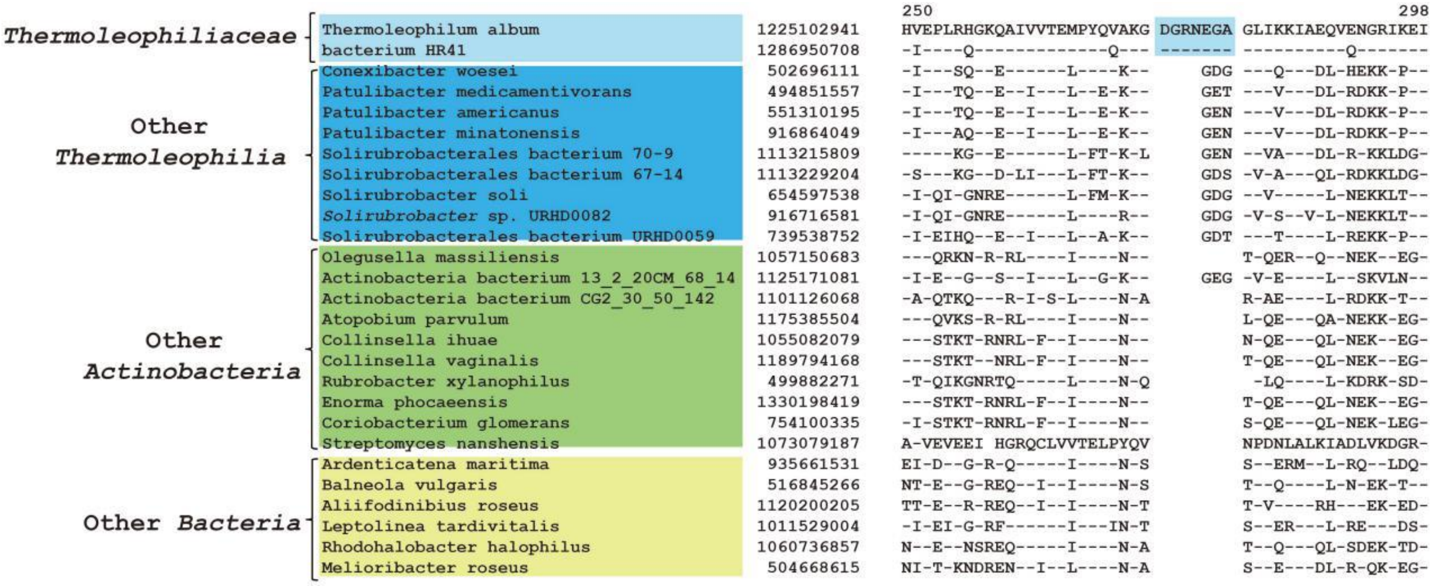


**Supplementary Figure S14.** *Thermoleophilaceae* family specific CSI. Partial alignment of the protein DNA gyrase subunit A showing a 8aa insertion that is specific to the family *Thermoleophilaceae*.

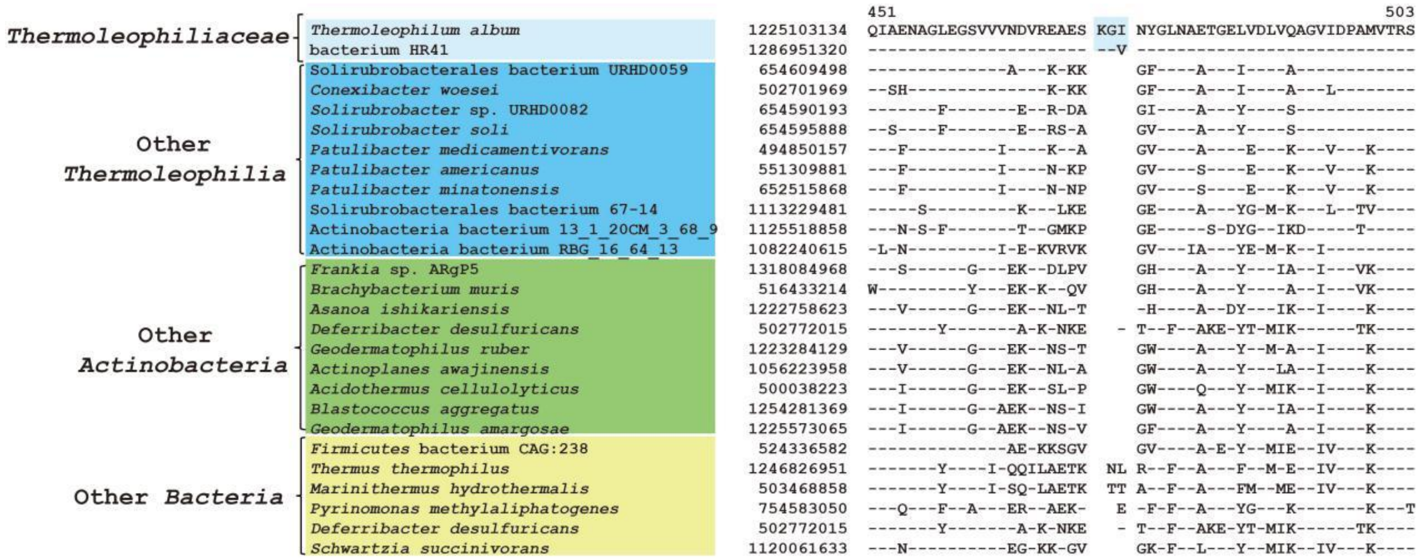


**Supplementary Figure S15.** *Thermoleophilaceae* family specific CSI. Partial alignment of the protein chaperonin GroEL showing a 3aa insertion that is specific to the family *Thermoleophilaceae*.

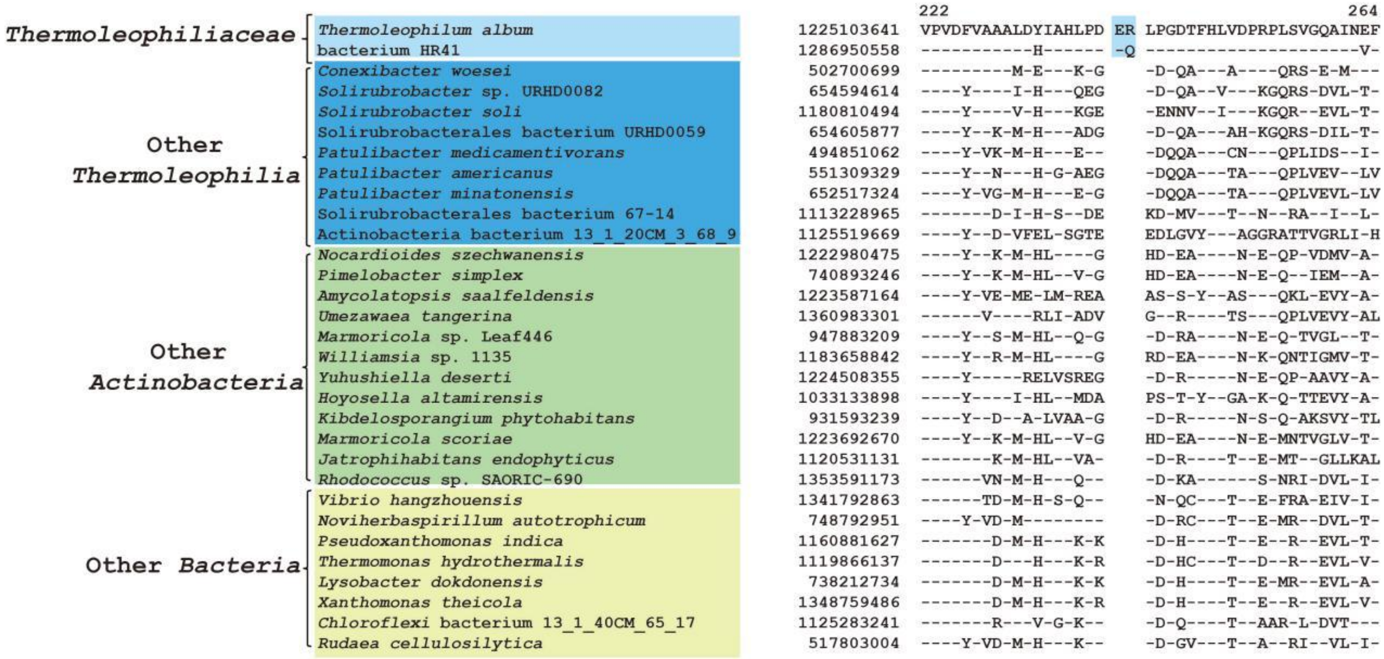


**Supplementary Figure S16.** *Thermoleophilaceae* family specific CSI. Partial alignment of the protein short chain dehydrogenase showing a 2aa insertion that is specific to the family *Thermoleophilaceae.*

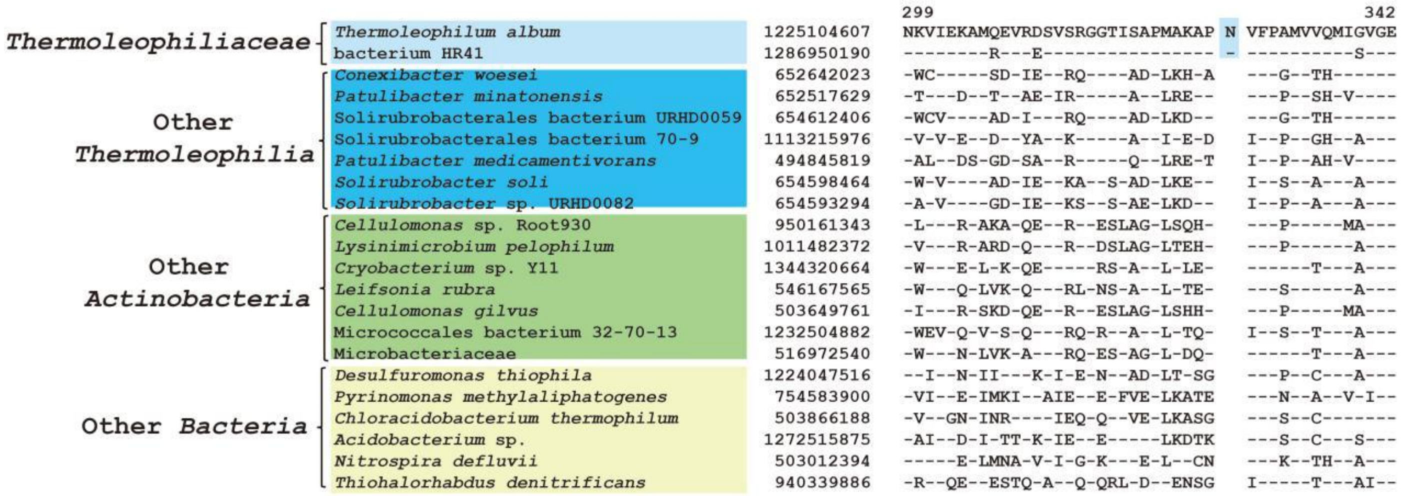


**Supplementary Figure S17.** *Thermoleophilaceae* family specific CSI. Partial alignment of the type II secretion system F family protein showing a 1aa insertion that is specific to the family *Thermoleophilaceae.*

| Group | Species | Accession | 429 | | 469 |
|---|---|---|---|---|---|
| *Thermoleophiliaceae* | *Thermoleophilum album* | 1093217654 | RLRDWLISRQRYWGCPIPVVYCE | R | ECGIVPVPDEQLPVELP |
| | bacterium HR41 | 1286950614 | ----------------------- | - | ----------------- |
| Other *Thermoleophilia* | Solirubrobacterales bacterium URHD0059 | 654612357 | ------L-----------IIH-D | | K--M-----D----V-- |
| | *Patulibacter medicamentivorans* | 494845103 | ------V-----------I-H-D | | R--V----ED----V-- |
| | Solirubrobacterales bacterium 67-14 | 1113226510 | ------V-------A---I---D | | ---M----ED----R-- |
| | *Patulibacter americanus* | 551307861 | ------V-------------H-P | | -H------ED----V-- |
| | *Conexibacter woesei* | 652642365 | ------L-----------I-H-P | | RD-M-----D----V-- |
| | *Patulibacter minatonensis* | 652518388 | ------V------------IH-P | | -H-M----ED----V-- |
| | *Solirubrobacter* sp. URHD0082 | 654593141 | Y-----L-----------I---- | | -H-M----EVD---V-- |
| | *Solirubrobacter soli* | 1180812565 | -GG-FP-----A--T---I-H-- | | A--T----LD----R-- |
| | Solirubrobacterales bacterium 70-9 | 1113217351 | -AN-FS-----S--T---I-H-P | | D--P----EAD---V-- |
| | Actinobacteria bacterium 13_1_20CM_3_68_9 | 1125520100 | GNV--AL--E----T-L-IWE-A | | SE-CEERFCAGSVA--R |
| Other *Actinobacteria* | *Planomonospora sphaerica* | 1057616277 | ------L----F------IIH-V | | D--E----------T-- |
| | *Mycetocola* sp. CGMCC 1.16372 | 1383223523 | --------------T---IIH-- | | K--E----ED---I--- |
| | *Slackia piriformis* | 496429541 | --------------N---AI--D | | --------E-D------ |
| | *Propionicicella superfundia* | 655300646 | ------L----F------IIH-P | | A--E------------- |
| | Actinobacteria bacterium IMCC26207 | 918748503 | ------L----F------I---D | | A-------ED----LA- |
| | *Lysinimicrobium aestuarii* | 1011403761 | --------------T---IIH-- | | T--E-----D----T-- |
| | *Tessaracoccus massiliensis* | 749688402 | ------L----F-------IH-D | | K--E-----D----R-- |
| | *Streptomyces roseochromogenus* | 559016619 | ------L-------T---II--D | | S--A-----D----K-- |
| | *Demequina lutea* | 1011153759 | --------------T---I-H-- | | A--E-----D----T-- |
| | *Nocardiopsis flavescens* | 1120521595 | ------L-------T---I-H-P | | A--Q----------T-- |
| | *Lysinimicrobium pelophilum* | 1011482953 | --------------T---I-H-D | | D--E-----D----V-- |
| | *Nocardioides* sp. Leaf307 | 948246279 | ------L----F------IIH-P | | S--E----ED------- |
| | *Actinomadura meyerae* | 1219311358 | ------V----F------I-H-P | | S--E----------T-- |
| | *Eggerthella* sp. CAG:209 | 524383414 | --------------N---I---D | | D-------ED----T-- |
| Other *Bacteria* | *Clostridium tyrobutyricum* | 746631014 | ------V-------T-------- | | K--V----E-------- |
| | *Synechococcus* sp. PCC 7336 | 515894914 | -------------------IH-P | | D---------------- |
| | *Saccharomonospora cyanea* | 491600532 | Y-----L-----------I---D | | S--V----E-S------ |
| | *Leucothrix mucor* | 551332762 | -----GV-----------II--D | | ---A-----D------- |
| | Proteobacteria bacterium | 1273645307 | -----GV-----------II--D | | D--A-----D------- |
| | *Desnuesiella massiliensis* | 944178210 | --------------A---II--D | | H--T----E-------- |
| | *Hathewaya proteolytica* | 1120028984 | ------V-------A-------D | | K-------ED----K-- |
| | Bacillaceae bacterium G1 | 1104311074 | -I----------------I---D | | R-------K-----L-- |
| | *Phaeomoniella chlamydospora* | 821070414 | --------------T---IIH-N | | S--A--I---------- |
| | *Tessaracoccus oleiagri* | 1086274802 | ------L----F------I-H-- | | Q--E----------R-- |
| | *Hyphomicrobium sulfonivorans* | 1057056127 | -----GV-------------H-- | | T--------KD------ |
| | *Meiothermus timidus* | 648543182 | --------------T---MIH-D | | R-------Y-------- |

**Supplementary Figure S18.** *Thermoleophilaceae* family specific CSI. Partial alignment of the protein leucyl-tRNA synthetase showing a 1aa insertion that is specific to the family *Thermoleophilaceae.*

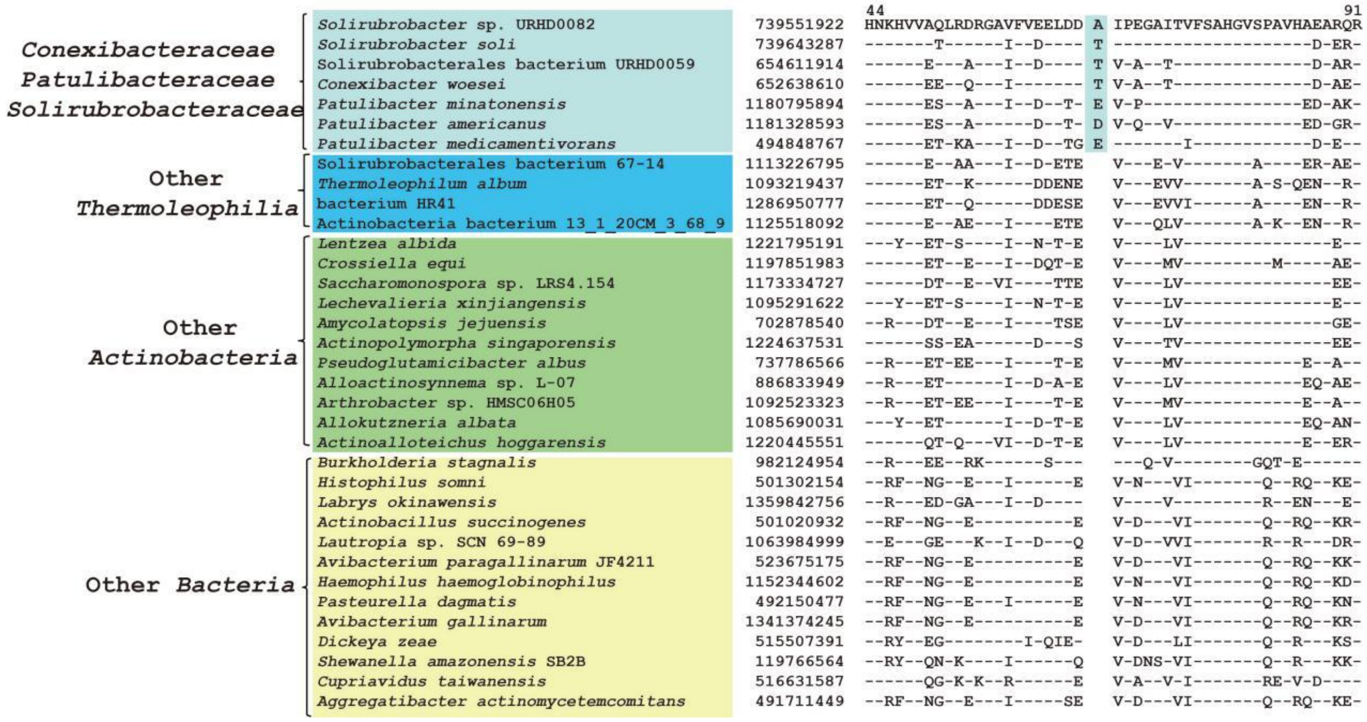


**Supplementary Figure S19.** CSI specific to *Conexibacteraceae*, *Solirubrobacteraceae* and *Patulibacteraceae.* A 1aa insertion in the protein 4-hydroxy-3-methylbut-2-enyl diphosphate reductase that is uniquely shared by *Conexibacteraceae*, *Solirubrobacteraceae* and *Patulibacteraceae.*

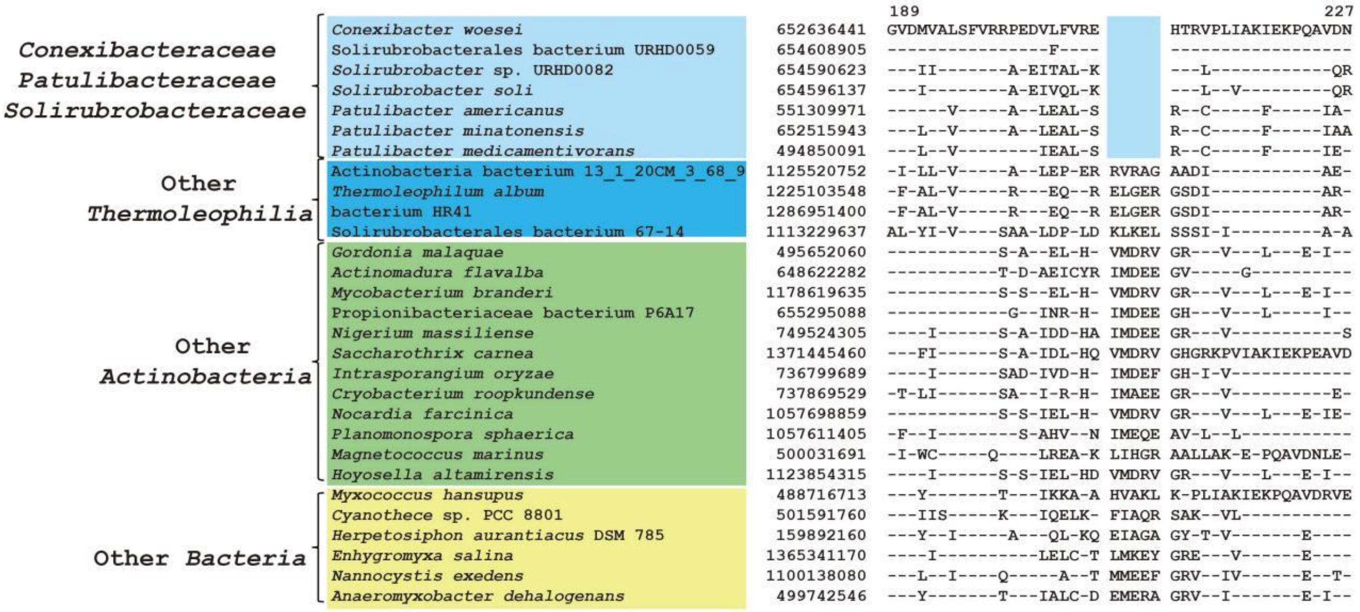


**Supplementary Figure S20.** CSI specific to *Conexibacteraceae, Solirubrobacteraceae* and *Patulibacteraceae*. A 5aa deletion in the protein pyruvate kinase that is uniquely shared by *Conexibacteraceae, Solirubrobacteraceae* and *Patulibacteraceae.*

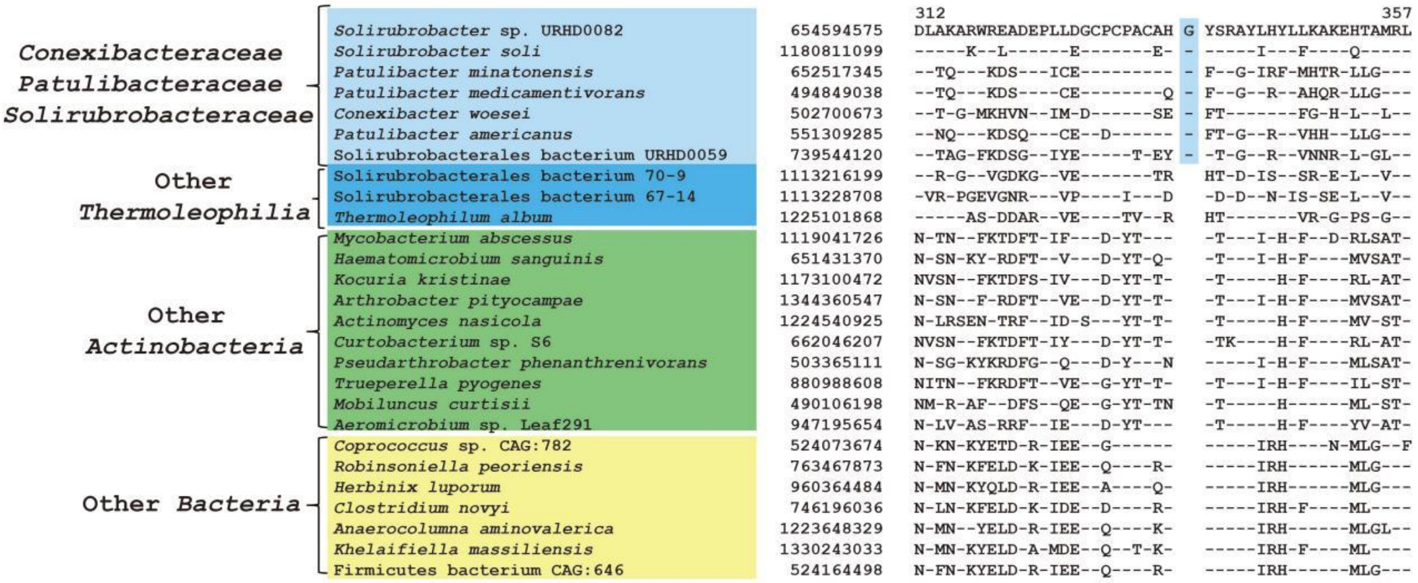


**Supplementary Figure S21.** CSI specific to *Conexibacteraceae*, *Solirubrobacteraceae* and *Patulibacteraceae*. A 1aa insertion in the protein tRNA-guanine (34) transglycosylase that is uniquely shared by *Conexibacteraceae*, *Solirubrobacteraceae* and *Patulibacteraceae*

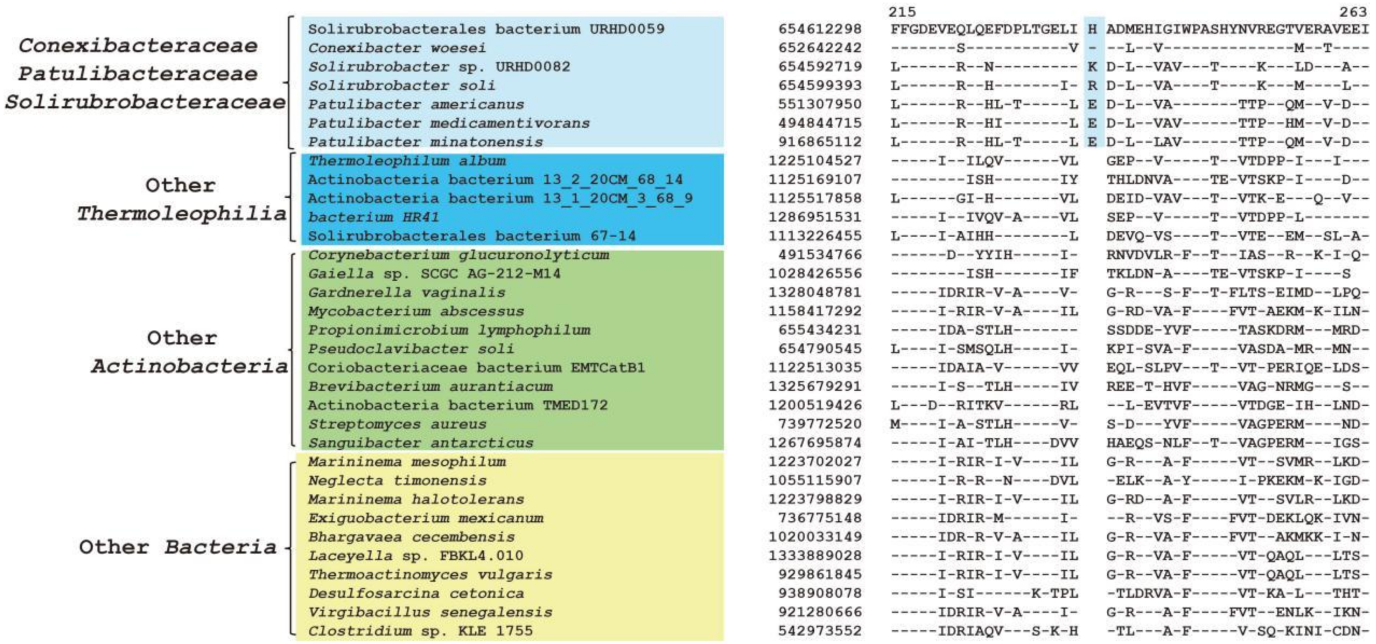


**Supplementary Figure S22.** CSI specific to *Conexibacteraceae*, *Solirubrobacteraceae* and *Patulibacteraceae*. A 1aa insertion in the protein excinuclease ABC subunit UvrB that is uniquely shared by *Conexibacteraceae*, *Solirubrobacteraceae* and *Patulibacteraceae*

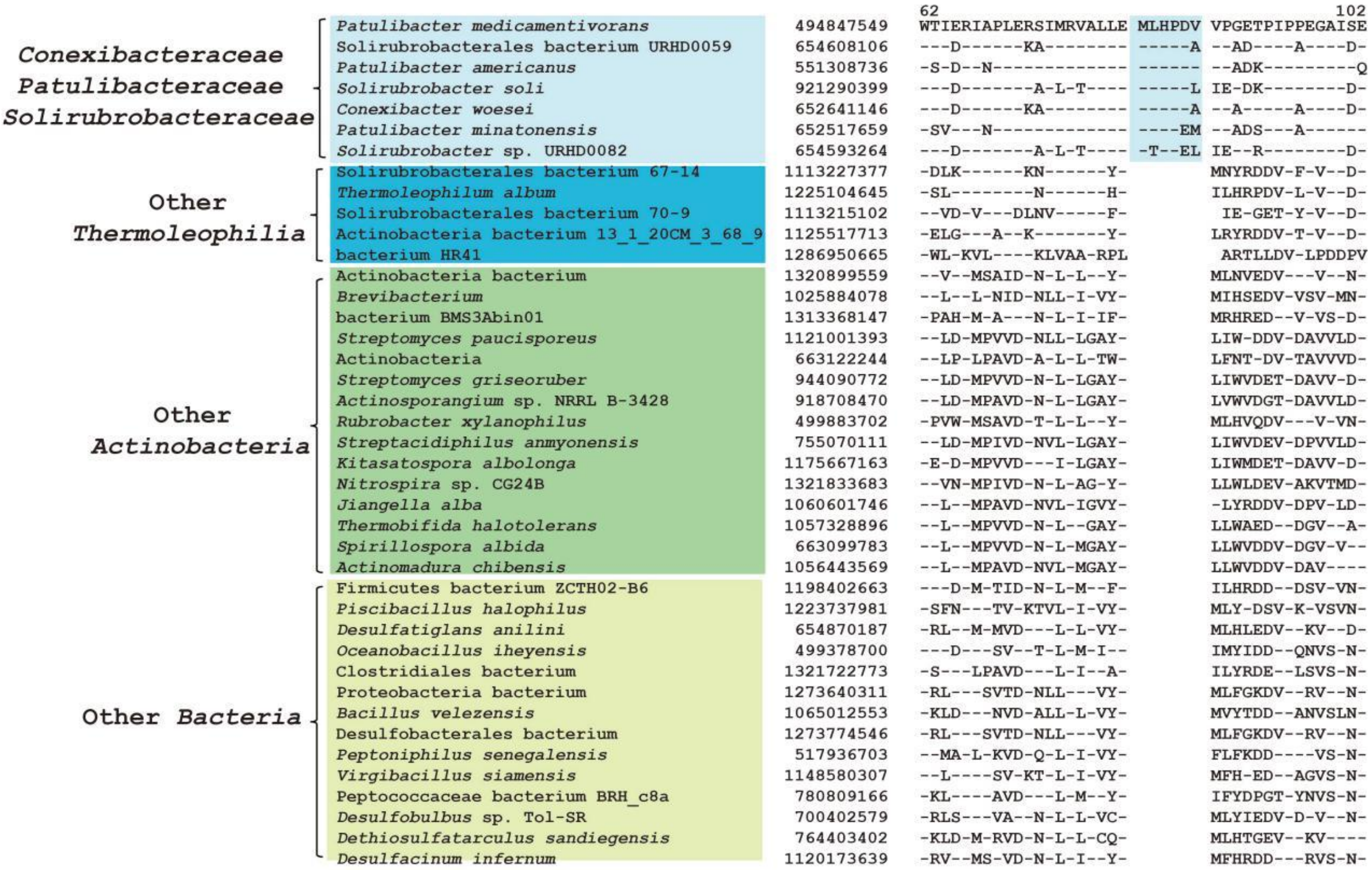


**Supplementary Figure S23.** CSI specific to *Conexibacteraceae, Solirubrobacteraceae* and *Patulibacteraceae*. A 6aa insertion in the protein transcription antitermination factor NusB that is uniquely shared by *Conexibacteraceae, Solirubrobacteraceae* and *Patulibacteraceae*

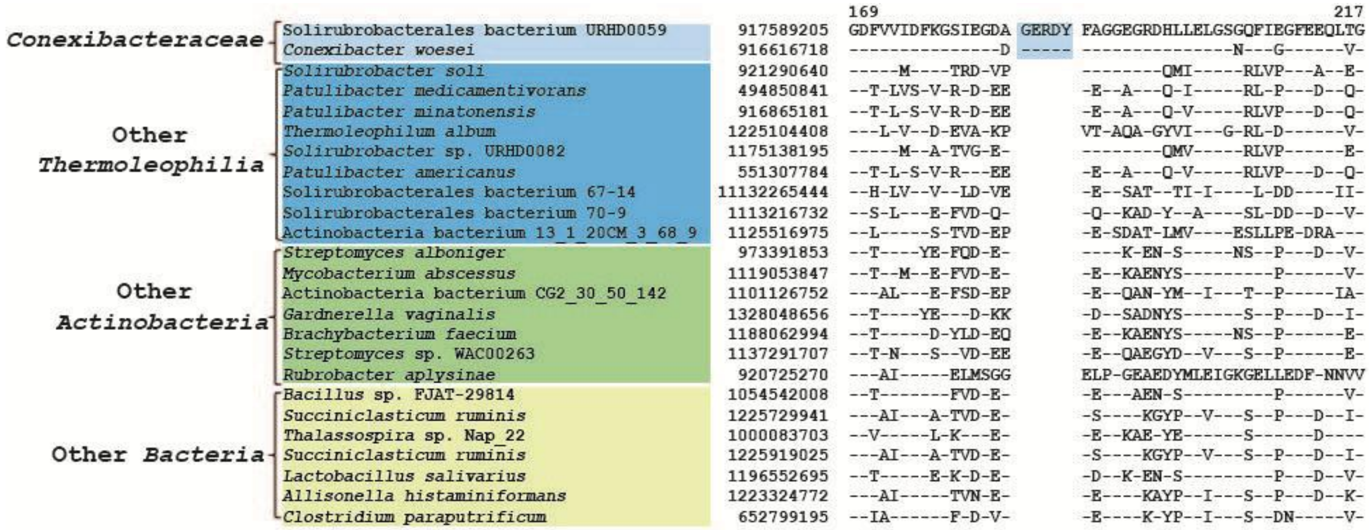


**Supplementary Figure S24.** *Conexibacteraceae* family specific CSI. Partial alignment of the protein trigger factor showing a 5aa insertion that is specific to the family *Conexibacteraceae.*

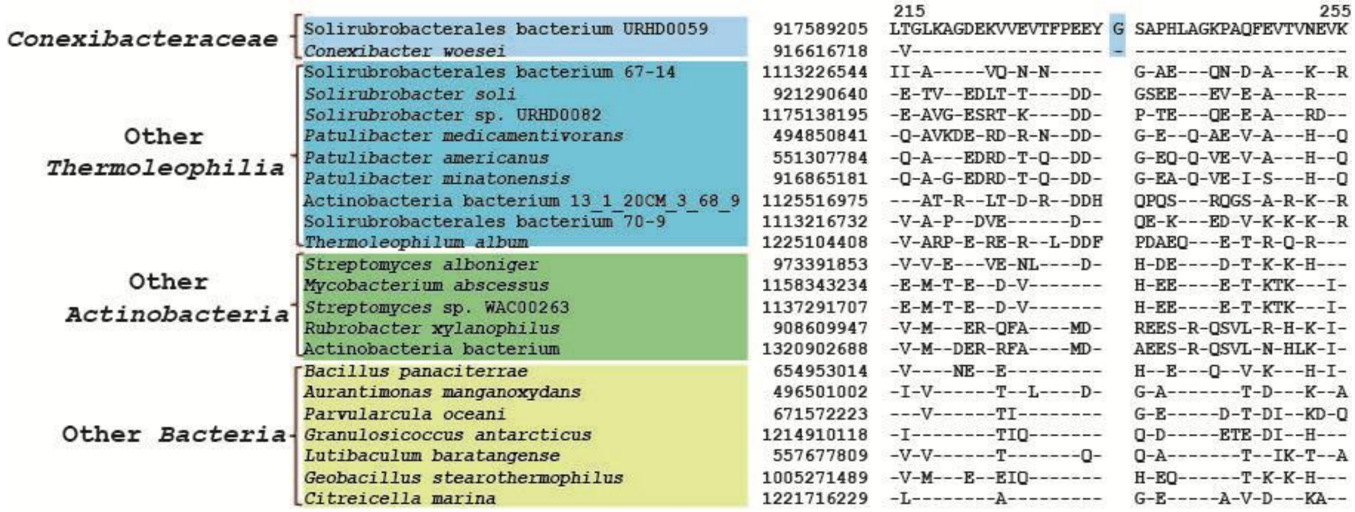

**Supplementary Figure S25.** *Conexibacteraceae* family specific CSI. Partial alignment of the protein trigger factor showing a 1aa insertion that is specific to the family *Conexibacteraceae.*

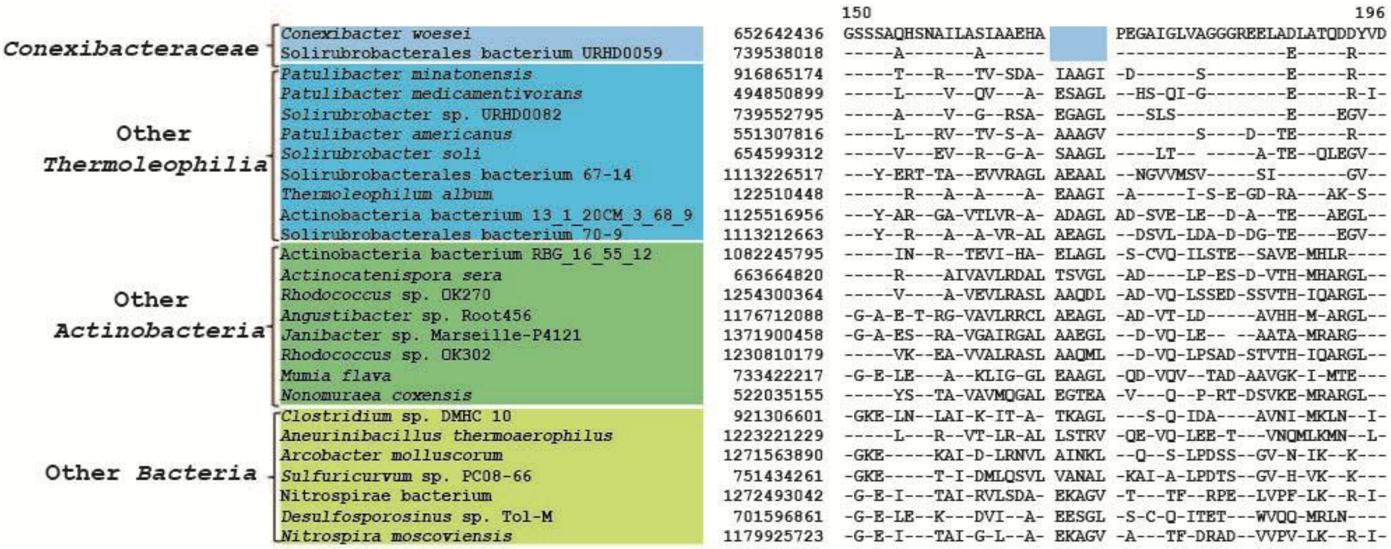


**Supplementary Figure S26.** *Conexibacteraceae* family specific CSI. Partial alignment of the protein glutamate-5-semialdehyde dehydrogenase showing a 5aa deletion that is specific to the family *Conexibacteraceae.*

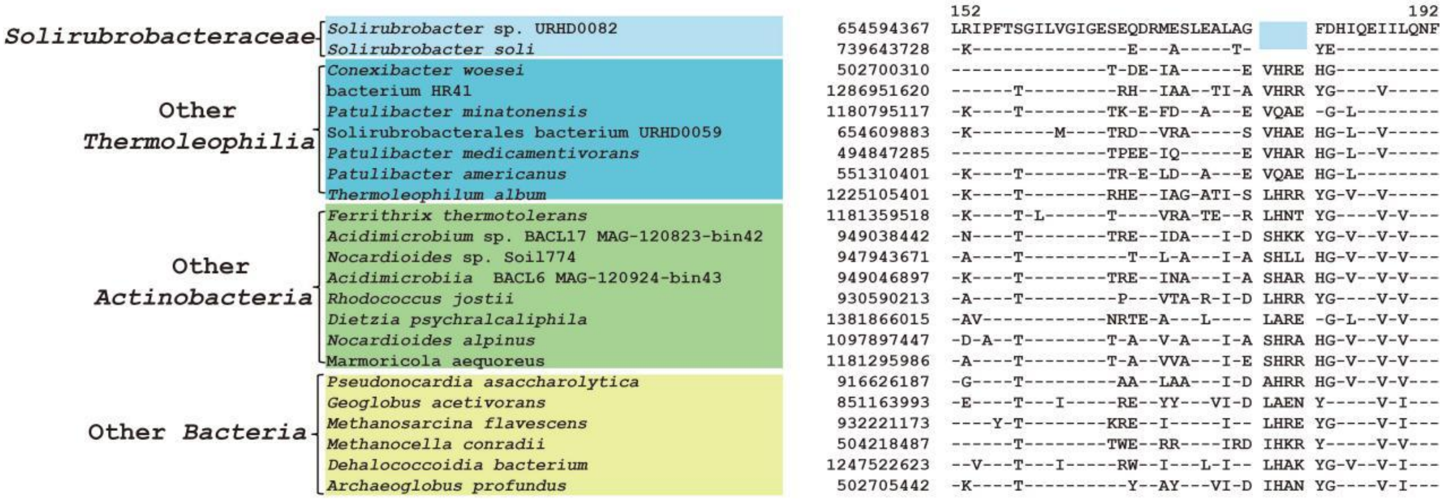


**Supplementary Figure S27.** *Solirubrobacteraceae* family specific CSI. Partial alignment of the protein 7,8-didemethyl-8-hydroxy-5-deazariboflavin synthase subunit CofH showing a 4aa deletion that is specific to the family *Solirubrobacteraceae.*

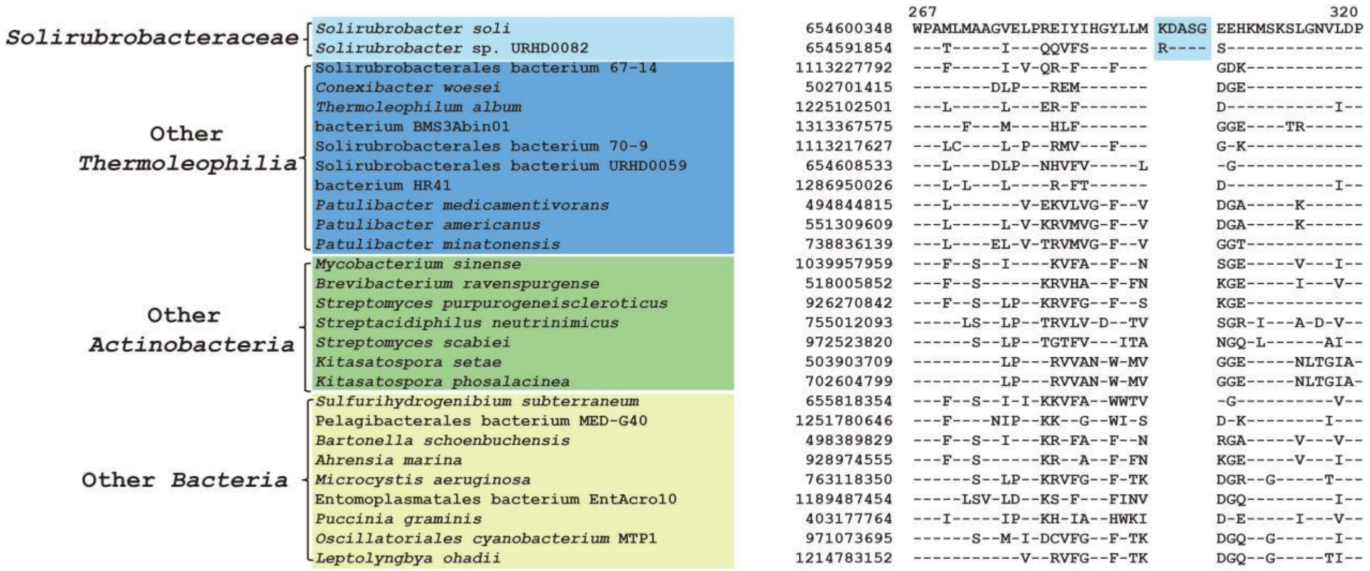


**Supplementary Figur28.** *Solirubrobacteraceae* family specific CSI. Partial alignment of the protein methionine--tRNA ligase showing a 5aa insertion that is specific to the family *Solirubrobacteraceae.*

| Group | Species | Accession | 20 | | 65 |
|---|---|---|---|---|---|
| *Solirubrobacteraceae* | *Solirubrobacter soli* | 654597239 | DEEVERFGGELSKVLDHIELIGEL | G | DLADVPPTSHVIDVENALRAD |
| | *Solirubrobacter* sp. URHD0082 | 654591156 | E--I---S--------YV-T-E-- | - | G-D--------V--------- |
| Other *Thermoleophilia* | *Thermoleophilum album* | 1225105051 | -------TR--GTI-E-V-R-QQ- | | --EE-------VAL--V--P- |
| | bacterium HR41 | 1286951430 | -------TR--GTI-E-V-R-QQ- | | ---G-E-----VELV-V--P- |
| | *Conexibacter woesei* | 502699726 | -D--DAMAR---A----V-R---- | | E-D---A----V--V----P- |
| | Solirubrobacterales bacterium URHD0059 | 917590160 | GD--GKMAA---S--G---K---- | | T-D--A-----VE-A----E- |
| | *Patulibacter americanus* | 551308586 | -D-I-PMAR---A----L-T---- | | --EG-E--A--LAERSP---- |
| | Solirubrobacterales bacterium 67-14 | 1113227296 | E--IDTLT----S----VDKLA-V | | -IEG-E-----VPL--V--D- |
| | *Patulibacter medicamentivorans* | 494844308 | ED-I-PMAR---A----L-T---- | | --DG-A--A--LAS-SR---- |
| | *Patulibacter minatonensis* | 652518057 | -D-I-PMAR---A--G-L-T---- | | --DG-E--A--LVERS----- |
| | Solirubrobacterales bacterium 70-9 | 1113214505 | -A---TMA----GI-E-VDR-SD- | | --EG-EA----VQL--VF--- |
| Other *Actinobacteria* | *Rubrobacter radiotolerans* | 740895524 | ------M--Q-GAI--S--R-Q-- | | --EG----ANPLNLT-VM-P- |
| | *Rubrobacter xylanophilus* | 499883081 | ----A-M--Q-GAI--S--K-R-- | | --EG----ANPLNLT-VF-P- |
| | Actinobacteria bacterium | 1320898905 | -----KMSEQ-GAI--S--E-R-- | | --E-----ASLL-PT-V--P- |
| | *Modestobacter marinus* | 504555336 | ---LD--A-Q-DQ--AAVARV--A | | AV-----M--AVPLT-V---- |
| | Actinobacteria bacterium | 1320901321 | -S---KMS-Q-GAI--S--Q-Q-- | | ---------NPMNFT-V--P- |
| | *Modestobacter* sp. VKM Ac-2676 | 1137684693 | ---LD--A-Q-DQ--AAVARV--A | | GVG----MT-AVPLT-V---- |
| | *Microbacterium* sp. No. 7 | 938898514 | ------LT-Q--AIV-N-AKVSQV | | ATP---A---P-PL--VF--- |
| | *Geodermatophilus ruber* | 1223288589 | ---LDH-A-Q--A---AVAQV-KA | | -V------T-AVPLT-VA-P- |
| | *Modestobacter caceresii* | 738388662 | ---LD--A-Q-DQ--AAVARV--A | | AV-----MT-AVPLT-V--P- |
| | *Geodermatophilus tzadiensis* | 1361075739 | ---LD--A-Q-GA---AVAQVQRA | | -V---A--T-AVPLT-V---- |
| Other *Bacteria* | *Yuhushiella deserti* | 1224507813 | -D-LDT-A-Q-DQI--AVAKV--V | | AGE-------AVPLT-VF--- |
| | *Blastococcus* sp. DSM 44268 | 1223730808 | ---LDL-A-Q-GA---AVAQV-KA | | -V------T-AVPMT-VF-E- |
| | *Gracilibacillus orientalis* | 1223446619 | -----T-TQQ-GDIINYA--LN-- | | -TD--K--T--L-LK-VM-K- |
| | *Deferribacter desulfuricans* | 502773662 | ---I-K-T---N-I--Y-HKLN-- | | NTD--E-----L-IT-VF-D- |
| | Longimonas halophila | 1267199459 | ---K--LARDM-RI----DTLN-- | | -T-G---M--GVTR--VT-S- |
| | *Bacillus luciferensis* | 1207714561 | E--A-K-SKQ-GAI--FA-QL--- | | -TTN-K--T--LKMR-V--K- |
| | *Symbiobacterium thermophilum* | 1190804005 | E--IDA-A-Q-NRI-E-V-RMNQ- | | -V------Y--VTLQHPF-E- |
| | *Virgibacillus necropolis* | 1221131361 | ---A-K-TKQ---IIHYAD-LN-- | | -TD-IE--T--LETK-V--K- |

**Supplementary Figure S29.** *Solirubrobacteraceae* family specific CSI. Partial alignment of the protein Asp-tRNA (Asn)/Glu-tRNA (Gln) amidotransferase subunit GatC showing a 1 aa insertion that is specific to the family *Solirubrobacteraceae.*

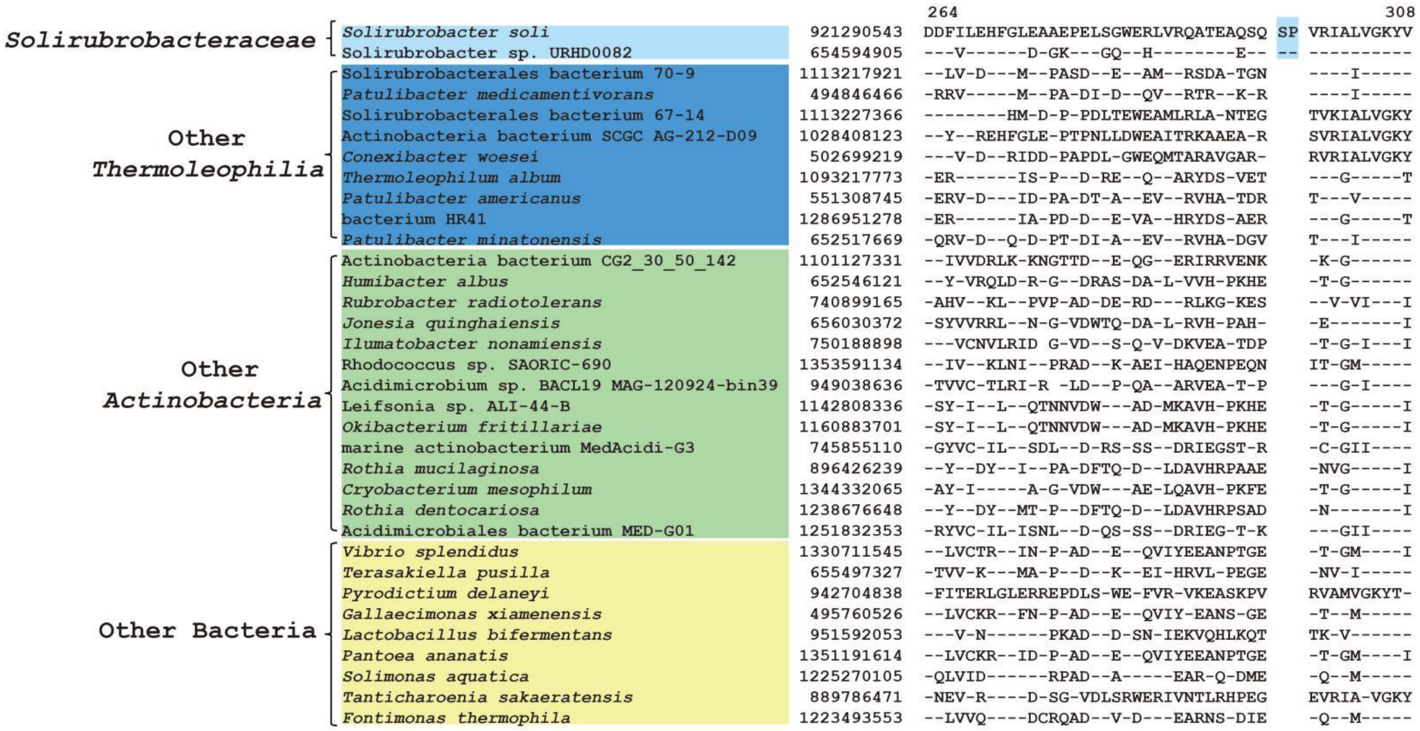


**Supplementary Figure S30.** *Solirubrobacteraceae* family specific CSI. Partial alignment of the protein CTP synthase showing a 2aa insertion that is specific to the family *Solirubrobacteraceae.*

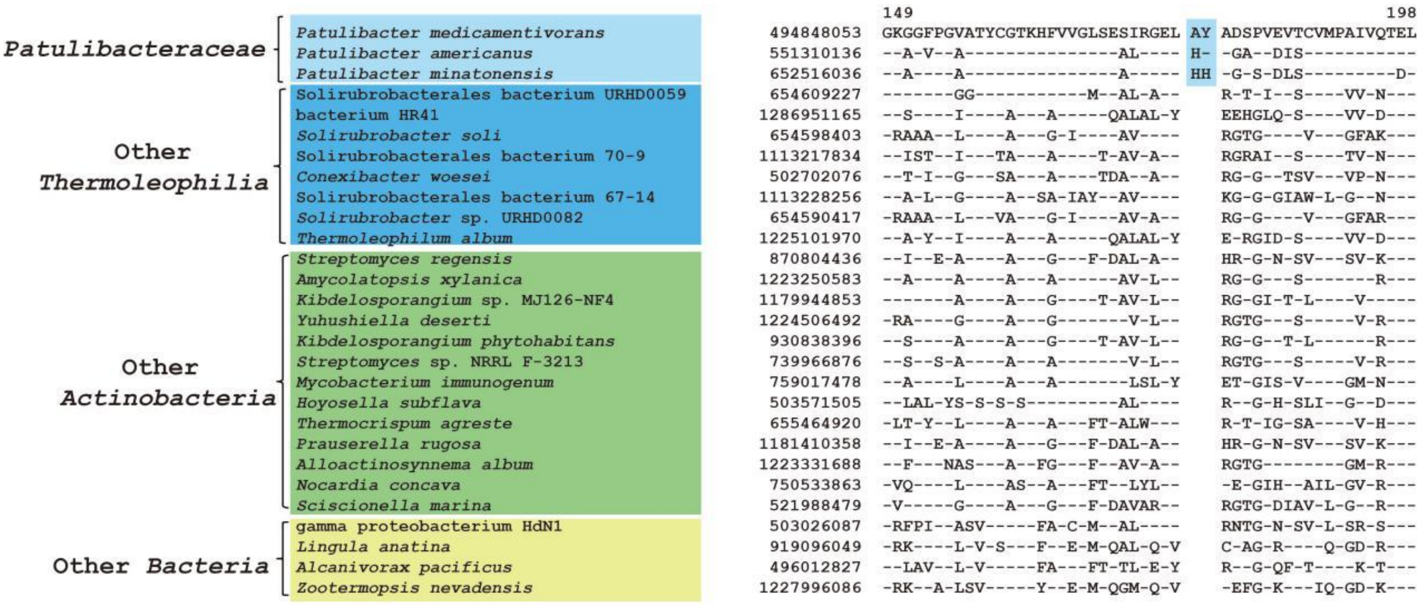


**Supplementary Figure S31.** *Patulibacteraceae* family specific CSI. Partial alignment of the protein SDR family NAD (P)-dependent oxidoreductase showing a 2aa insertion that is specific to the family *Patulibacteraceae*.

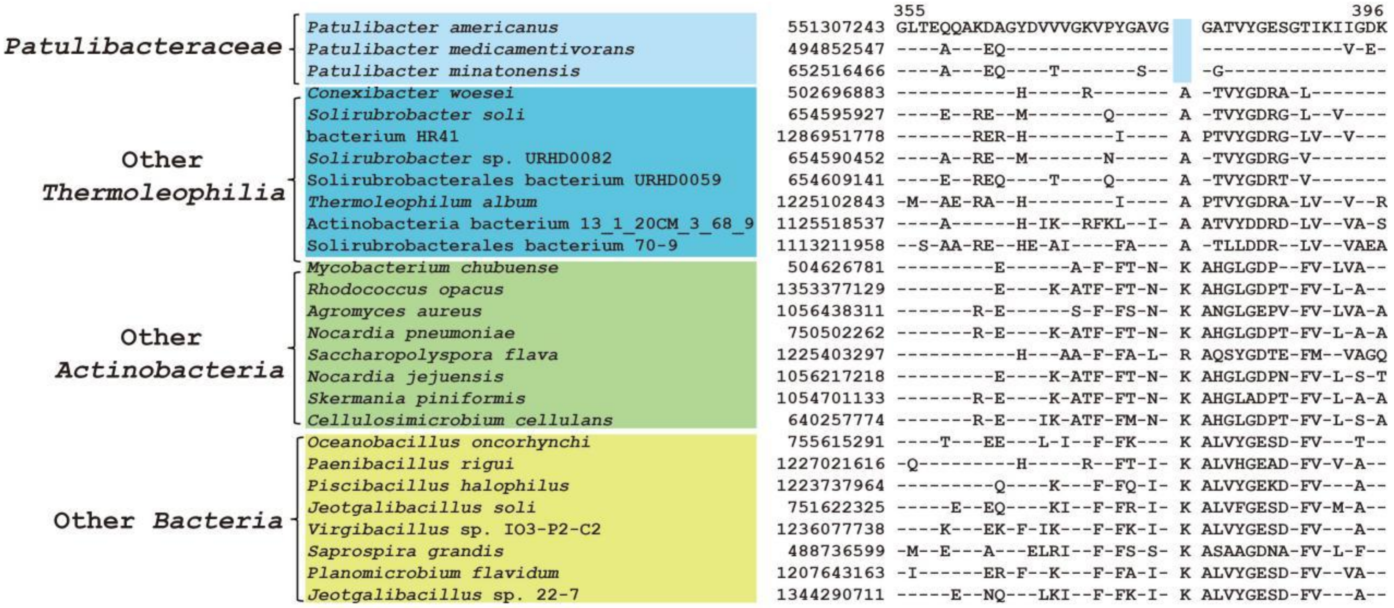


**Supplementary Figure S32.** *Patulibacteraceae* family specific CSI. Partial alignment of the protein dihydrolipoyl dehydrogenase showing a 1aa deletion that is specific to the family *Patulibacteraceae.*

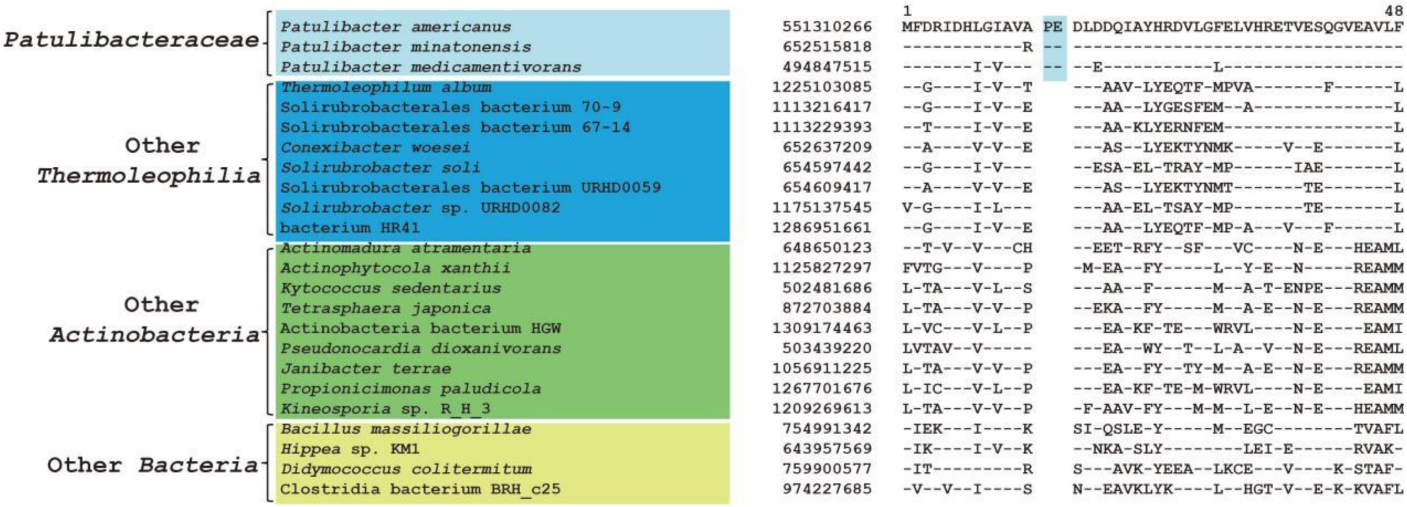


**Supplementary Figure S33.** *Patulibacteraceae* family specific CSI. Partial alignment of the protein methylmalonyl-CoA epimerase showing a 2aa insertion that is specific to the family *Patulibacteraceae.*

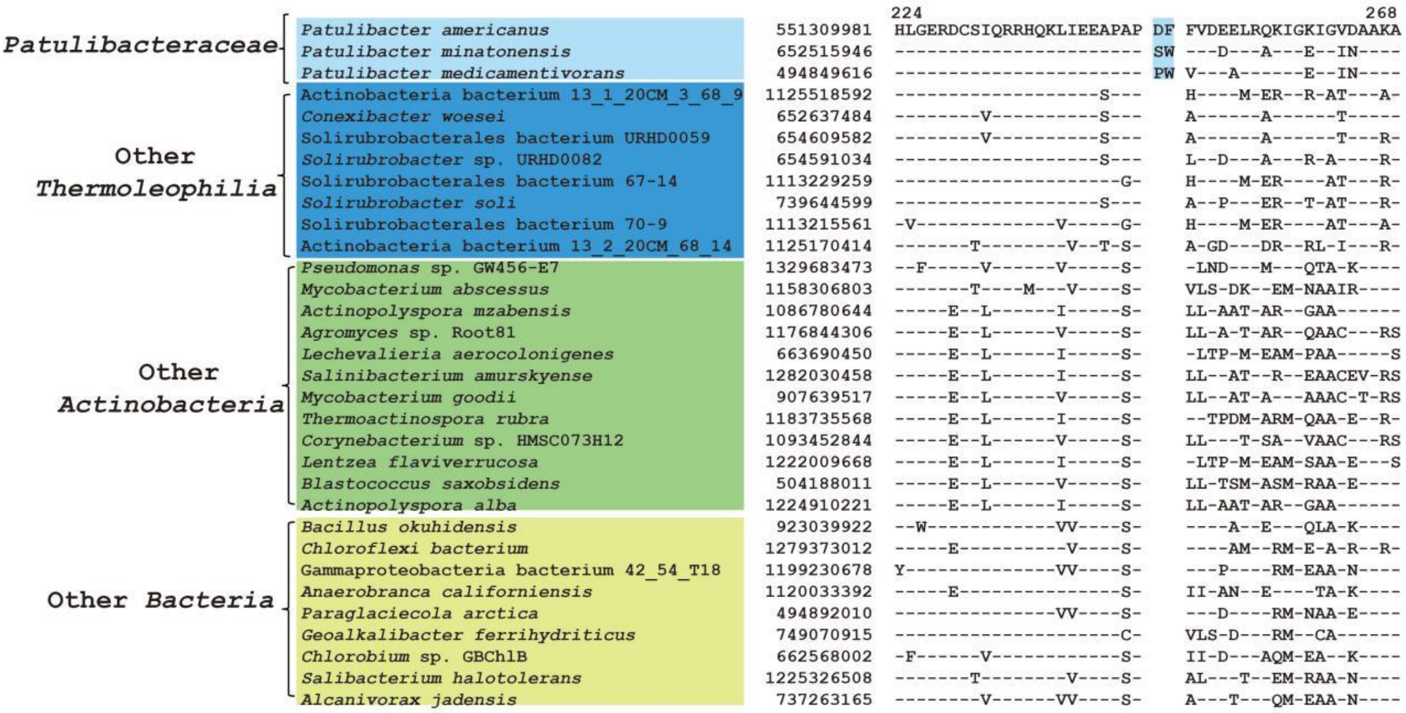


**Supplementary Figure S34.** *Patulibacteraceae* family specific CSI. Partial alignment of the protein acetyl-CoA carboxylase biotin carboxylase subunit showing a 2aa insertion that is specific to the family *Patulibacteraceae*.

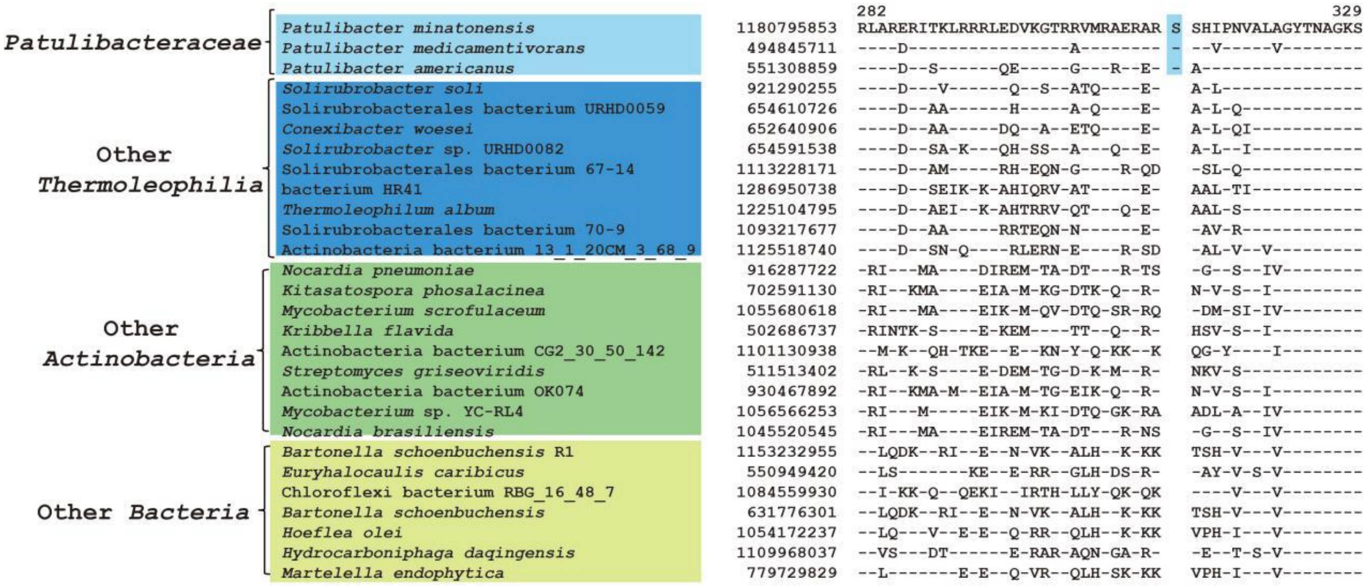


**Supplementary Figure S35.** *Patulibacteraceae* family specific CSI. Partial alignment of the protein GTPase HflX showing a 1aa insertion that is specific to the family *Patulibacteraceae*.

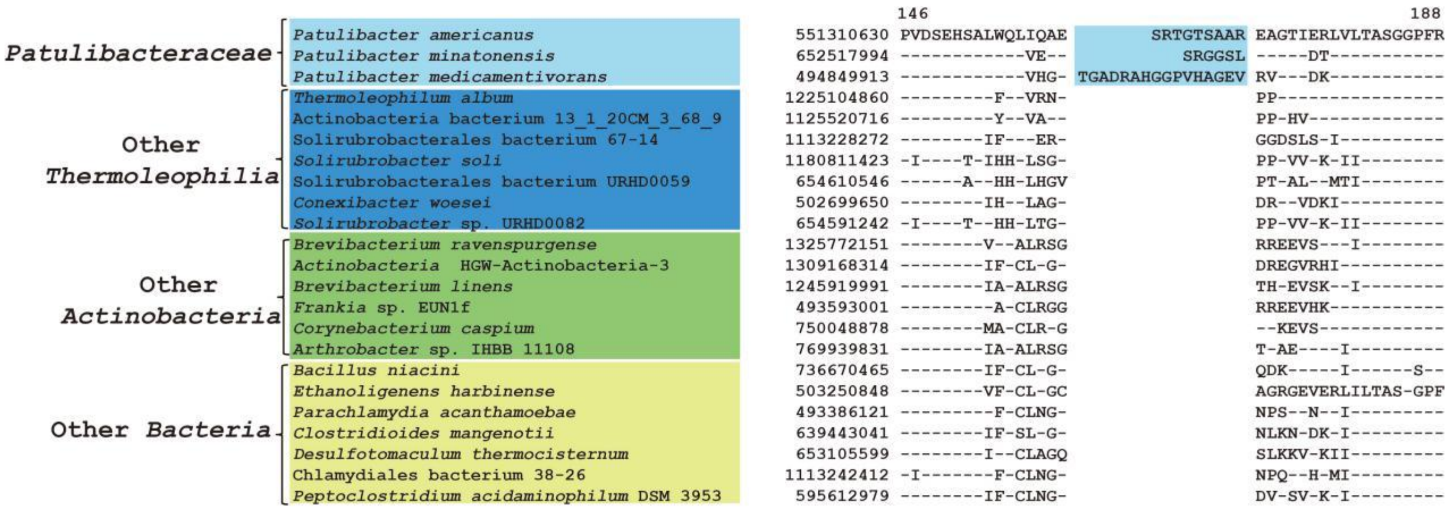


**Supplementary Figure S36.** *Patulibacteraceae* family specific CSI. Partial alignment of the protein 1-deoxy-D-xylulose-5-phosphate reductoisomerase showing a 6-8aa insertion that is specific to the family *Patulibacteraceae*

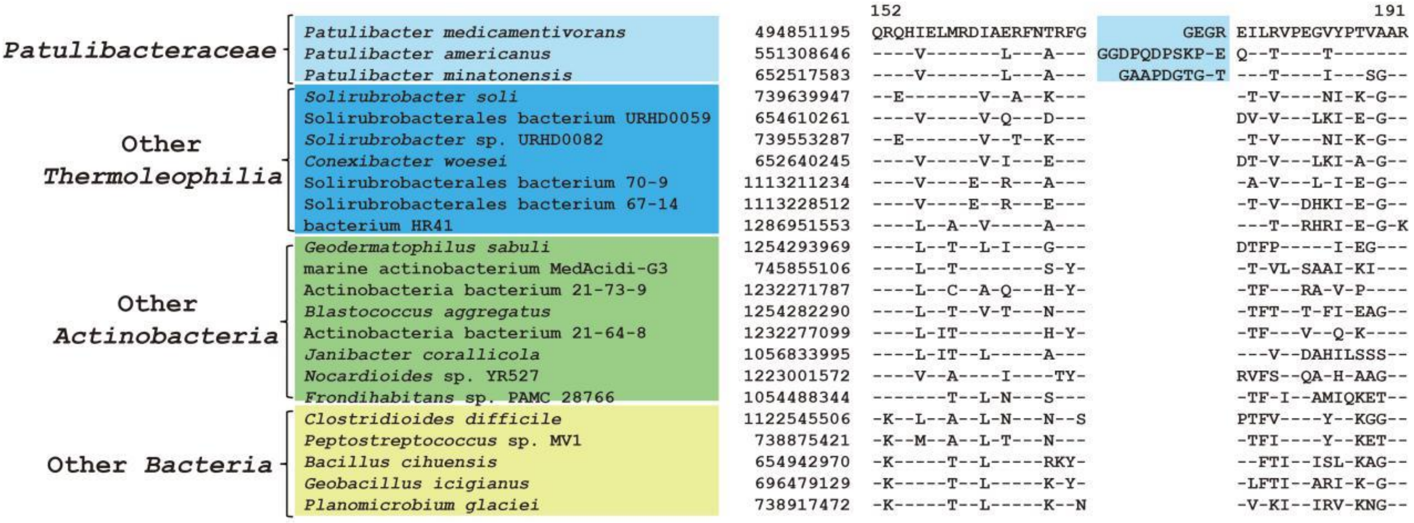


**Supplementary Figure S37.** *Patulibacteraceae* family specific CSI. Partial alignment of the protein tryptophan--tRNA ligase showing a 4-12aa insertion that is specific to the family *Patulibacteraceae*.

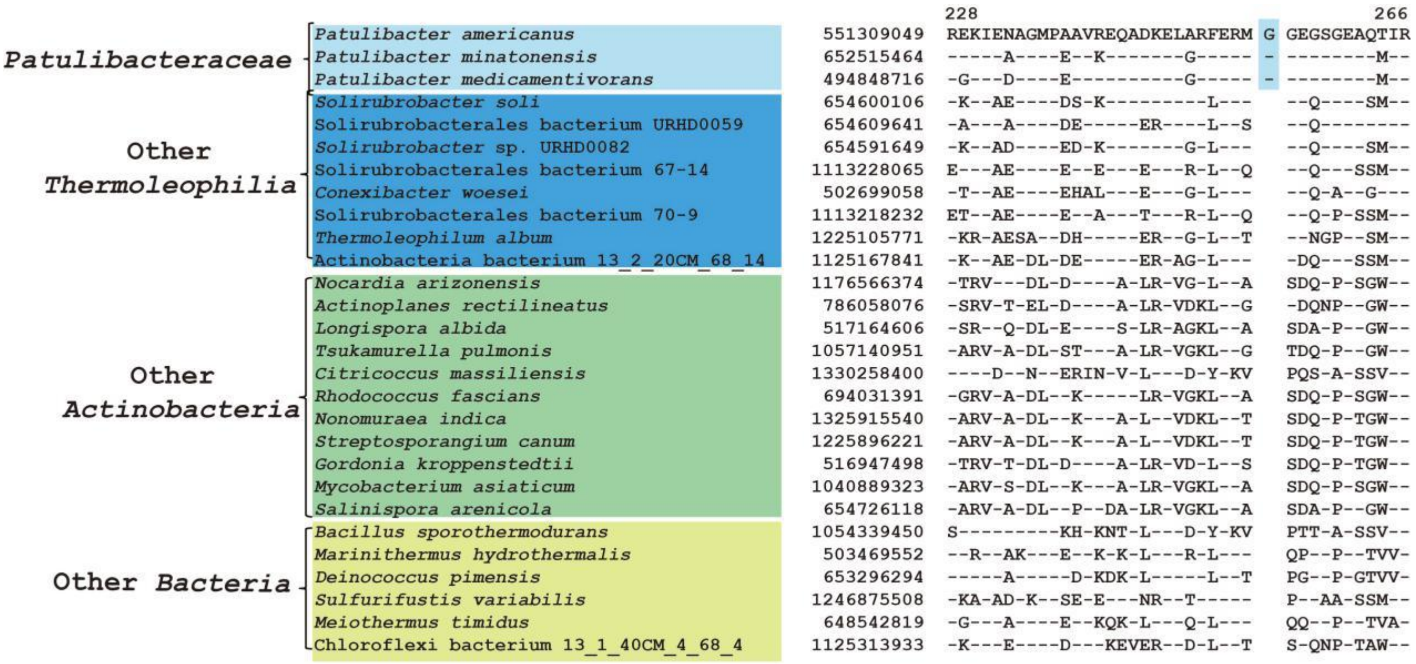


**Supplementary Figure S38.** *Patulibacteraceae* family specific CSI. Partial alignment of the protein endopeptidase La showing a 1aa insertion that is specific to the family *Patulibacteraceae.*

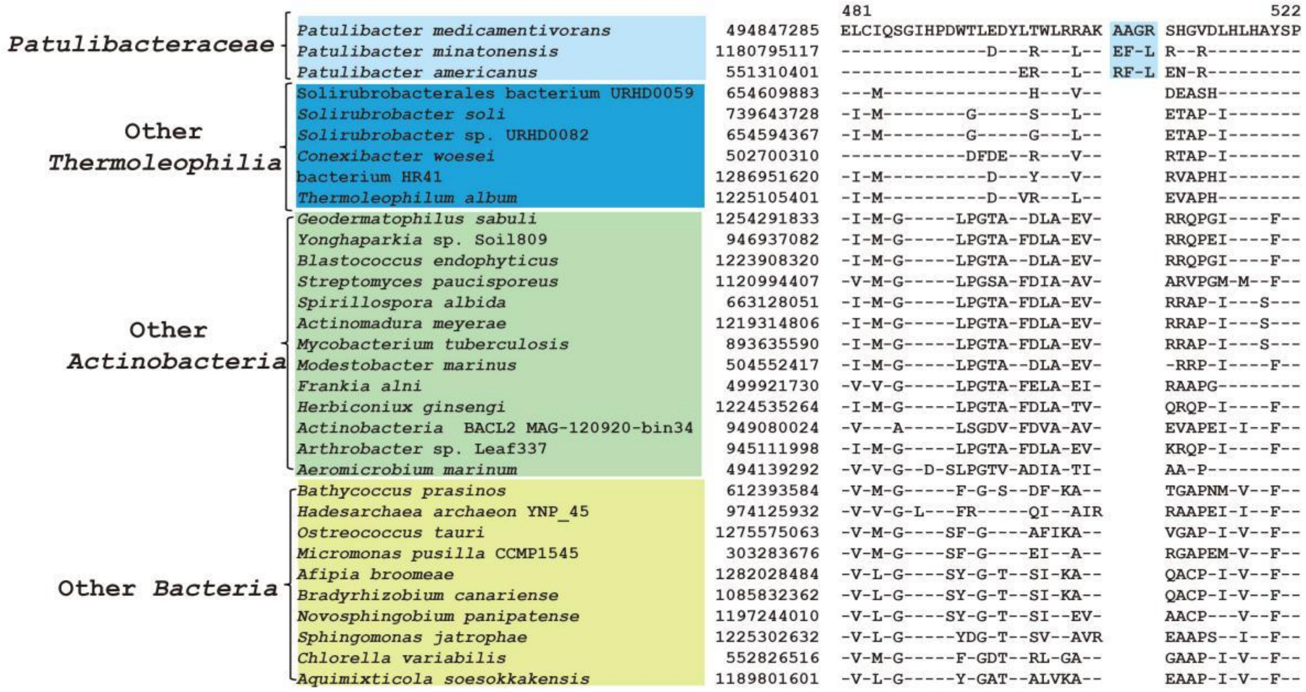


**Supplementary Figure S39.** *Patulibacteraceae* family specific CSI. Partial alignment of the protein 7,8-didemethyl-8-hydroxy-5-deazariboflavin synthase subunit CofH showing a 4aa insertion that is specific to the family *Patulibacteraceae.*

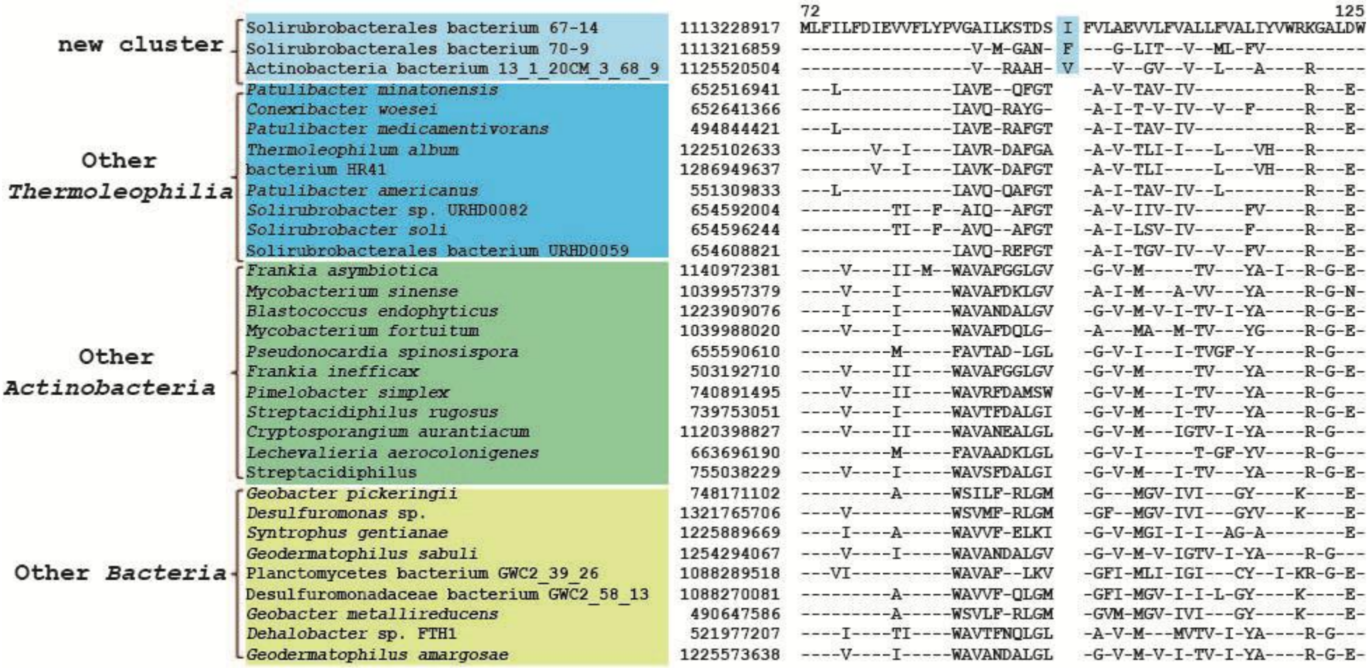


**Supplementary Figure S40. CSI specific to new cluster.** A 1aa insertion in the protein NADH-quinone oxidoreductase subunit I that is specific to the new cluster.

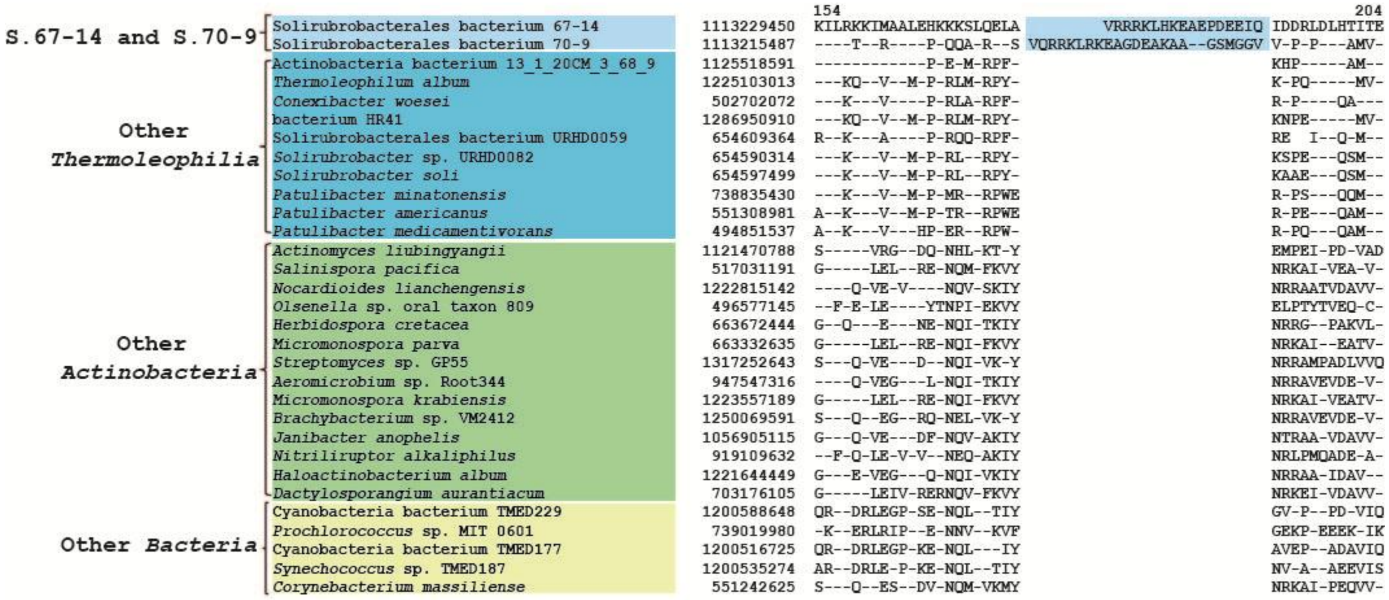


**Supplementary Figure S41.** CSI specific to Solirubrobacterales bacterium 67-14 and Solirubrobacterales bacterium 70-9. A 17-23aa insertion in the protein adenylosuccinate synthase that is uniquely shared by Solirubrobacterales bacterium 67-14 and Solirubrobacterales bacterium 70-9.

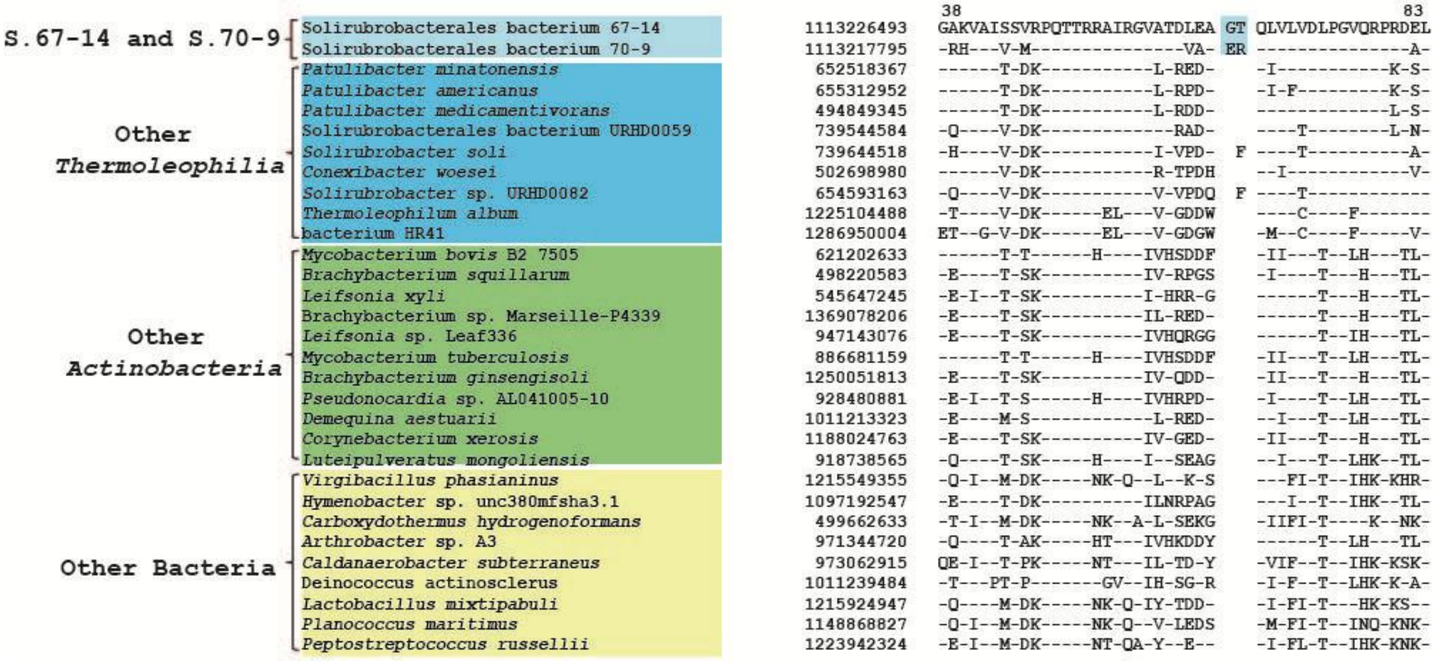

**Supplementary Figure S42.** CSI specific to Solirubrobacterales bacterium 67-14 and Solirubrobacterales bacterium 70-9. A 1-2aa insertion in the protein GTPase Era that is uniquely shared by Solirubrobacterales bacterium 67-14 and Solirubrobacterales bacterium 70-9.

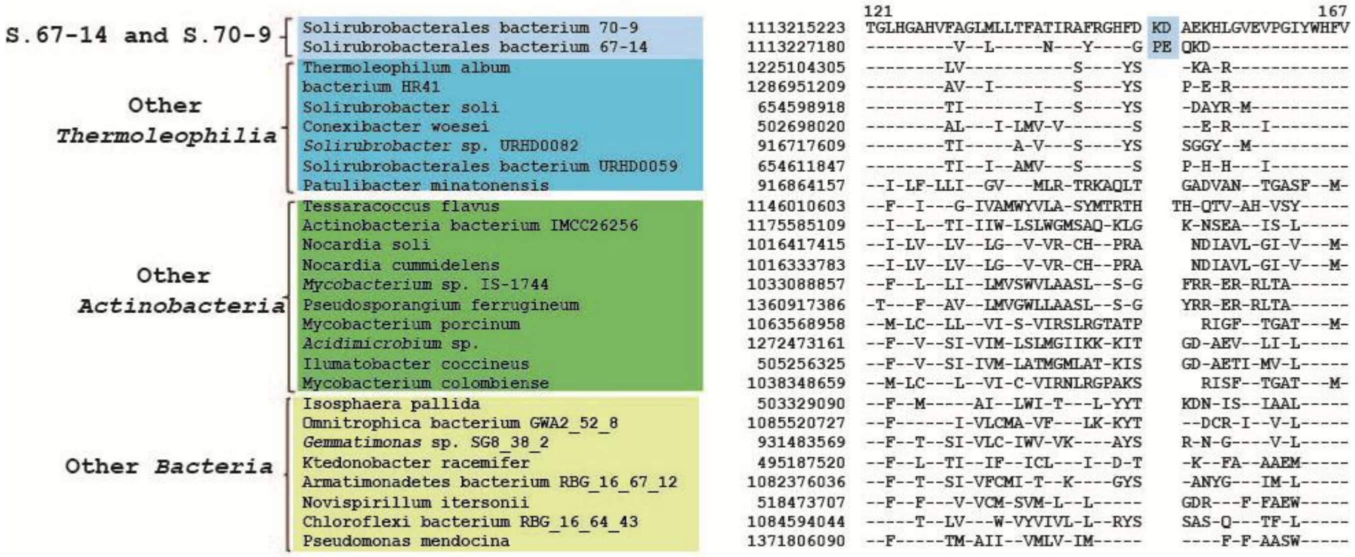


**Supplementary Figure S43.** CSI specific to Solirubrobacterales bacterium 67-14 and Solirubrobacterales bacterium 70-9. A 2aa insertion in the protein heme-copper oxidase subunit III that is uniquely shared by Solirubrobacterales bacterium 67-14 and Solirubrobacterales bacterium 70-9.

## Supplementary Table 1

## Summary of the species of the class *Thermoleophilia*

| Strain | length (Mb) | Contig-N50* | protein count | GC% | Accession No. | reference [PMID] |
|---|---|---|---|---|---|---|
| Actinobacteria bacterium 13_1_20CM_3_68_9 | 2.3 | 14,036 | 2144 | 69 | GCA_001920325.1 | 27843720 |
| bacterium HR41 | 1.6 | 6,277 | 1586 | 71 | GCA_002898855.1 | n.a |
| *Conexibacter woesei* DSM 14684 | 6 | 6,359,369 | 5609 | 71 | GCA_000025265.1 | 21304704 |
| *Gaiella occulta* F2-233 | 3.0 | 401,372 | 3119 | 72 | GCA_003351045.1 | n.a. |
| *Patulibacter americanus* DSM 16676 | 4.5 | 279,502 | 4003 | 74 | GCA_000420025.1 | 19126730 |
| *Patulibacter medicamentivorans* I11 | 5.1 | 24,679 | 4327 | 74 | GCA_000240225.2 | 23264500 |
| *Patulibacter minatonensis* DSM 18081 | 5.5 | 234,680 | 4891 | 74 | GCA_000519325.1 | 16449447 |
| *Solirubrobacter soli* DSM 22325 | 9.3 | 309,177 | 8365 | 72 | GCA_000423665.1 | 17625174 |
| *Solirubrobacter* sp. URHD0082 | 6.6 | 511,513 | 6197 | 72 | GCA_000425945.1 | n.a |
| Solirubrobacterales bacterium 67-14 | 3.2 | 90,498 | 2885 | 67 | GCA_001897355.1 | 26031303 |
| Solirubrobacterales bacterium 70-9 | 4.2 | 6,375 | 3408 | 69 | GCA_001898095.1 | 26031303 |
| Solirubrobacterales bacterium URHD0059 | 6.6 | 1,703,991 | 6170 | 72 | GCA_000688095.1 | n.a |
| *Thermoleophilum album* ATCC 35263 | 2.2 | 1,261,070 | 2047 | 69 | GCA_900108055.1 | 12710601 |

*: contig length at which 50% of total bases in assembly are in contigs of that length or greater, which defines assembly quality in terms of contiguity.

# Supplementary Table 2

## Details of the proteins used in phylogenomic analysis in Figure 1A

| protein name | length( aa)# | COG ID |
|---|---|---|
| Ribosome-binding ATPase YchF | 360 | COG0012* |
| Phenylalanine-tRNA ligase alpha subunit | 345 | COG0016* |
| Arginine-tRNA ligase | 549 | COG0018* |
| elongation factor Tu | 394 | COG0050 |
| 30S ribosomal protein S10 | 103 | COG0051 |
| 30S ribosomal protein S2 | 299 | COG0052* |
| Isoleucine-tRNA ligase 1 | 1040 | COG0060 |
| Phenylalanine-tRNA ligase beta subunit | 816 | COG0073 |
| 50S ribosomal protein L3 | 207 | COG0087* |
| 50S ribosomal protein L4 | 218 | COG0088* |
| 50S ribosomal protein L23 | 97 | COG0089 |
| 50S ribosomal protein L2 | 281 | COG0090* |
| 50S ribosomal protein L22 | 120 | COG0091* |
| 30S ribosomal protein S3 | 249 | COG0092* |
| 50S ribosomal protein L14 | 122 | COG0093* |
| 50S ribosomal protein L5 | 185 | COG0094* |
| 30S ribosomal protein S8 | 132 | COG0096* |
| 50S ribosomal protein L6 | 177 | COG0097* |
| 30S ribosomal protein S5 | 179 | COG0098* |
| Phosphoglycerate kinase | 395 | COG0126 |
| Serine-tRNA ligase | 425 | COG0172* |
| Aspartate-tRNA ligase | 596 | COG0173 |
| 30S ribosomal protein S15 | 89 | COG0184* |
| 30S ribosomal protein S19 | 90 | COG0185* |
| DNA gyrase subunit A | 842 | COG0188 |
| Guanylate kinase | 127 | COG0194 |
| 30S ribosomal protein S17 | 113 | COG0186* |
| Transcription termination/antitermination protein NusA | 398 | COG0195 |
| 50S ribosomal protein L16 | 139 | COG0197* |
| 50S ribosomal protein L24 | 103 | COG0198 |
| 50S ribosomal protein L15 | 158 | COG0200* |
| DNA-directed RNA polymerase subunit alpha | 313 | COG0202* |
| 50S ribosomal protein L17 | 95 | COG0203 |
| 50S ribosomal protein L27 | 84 | COG0211 |
| 50S ribosomal protein L7/L12 | 127 | COG0222 |
| 30S ribosomal protein S16 | 84 | COG0228 |
| Ribosome-recycling factor | 186 | COG0233 |
| 30S ribosomal protein S18 | 84 | COG0238 |
| 50S ribosomal protein L10 | 180 | COG0244 |
| 50S ribosomal protein L29 | 68 | COG0255 |

| | | |
|---|---|---|
| 50S ribosomal protein L18 | 115 | COG0256* |
| Elongation factor Ts | 262 | COG0264 |
| DNA ligase | 684 | COG0272 |
| Translation initiation factor IF-3 | 162 | COG0290 |
| 50S ribosomal protein L35 | 64 | COG0291 |
| 50S ribosomal protein L20 | 118 | COG0292 |
| 50S ribosomal protein L9 | 149 | COG0359 |
| 30S ribosomal protein S6 | 99 | COG0360 |
| molecular chaperone DnaK | 638 | COG0443 |
| DNA recombination and repair protein | 355 | COG0468 |
| CTP synthase | 546 | COG0504 |
| GTPase ObgE/CgtA | 430 | COG0536 |
| Signal recognition particle protein | 454 | COG0541* |
| Chromosomal replication initiator protein DnaA | 454 | COG0593 |

[#] Protein length is from *Thermoleophilum album* ATCC 35263.

*23 Proteins adopted from 40 universal, single-copy phylogenetic marker genes (*Nat Methods*. 2013 Sep;10(9):881-4).

## Supplementary Table 3

## Summary of 16S rRNA sequences used in phylogenetic tree

| Strain | Accession No. | length(bp) |
|---|---|---|
| *Conexibacter arvalis* KV-963 | AB597951.1 | 1487 |
| *Conexibacter woesei* DSM 14684 | NR_074830.1 | 1536 |
| *Gaiella occulta* F2-233 | NR_118138.1 | 1544 |
| *Gaiella* sp. EBR4-R2 | KC251738.1 | 1416 |
| *Gaiella* sp. EBR4-RS1 | KC251739.1 | 1410 |
| *Parviterribacter kavangonensis* D16/0/H6 | NR_148601.1 | 1456 |
| *Parviterribacter multiflagellatus* A22/0/F9_1 | NR_148602.1 | 1455 |
| *Patulibacter americanus* CP177-2 | AJ871306.1 | 1459 |
| *Patulibacter brassicae* SD | KT581436.1 | 1494 |
| *Patulibacter ginsengiterrae* P4-5 | NR_108221.1 | 1455 |
| *Patulibacter medicamentivorans* I11 | NR_121691.1 | 1541 |
| *Patulibacter minatonensis* KV-614 | AB193261.1 | 1528 |
| *Rubrobacter aplysinae* RV113 | NR_133849.1 | 1512 |
| *Rubrobacter bracarensis* VF70612_S5 | FR852392.1 | 1457 |
| *Rubrobacter calidifluminis* RG-1 | NR_125703.1 | 1474 |
| *Rubrobacter indioceani* SCSIO 08198 | MF919580.1 | 1372 |
| *Rubrobacter naiadicus* RG-3 | NR_125704.1 | 1473 |
| *Rubrobacter radiotolerans* P 1 | NR_029191.2 | 1532 |
| *Rubrobacter taiwanensis* LS-293 | NR_025220.1 | 1476 |
| *Rubrobacter xylanophilus* PRD-1 | NR_119187.1 | 1509 |
| *Solirubrobacter ginsenosidimutans* BXN5-15 | NR_108192.1 | 1489 |
| *Solirubrobacter pauli* B33D1 | NR_042722.1 | 1366 |
| *Solirubrobacter phytolaccae* GTGR-8 | NR_133858.1 | 1432 |
| *Solirubrobacter soli* Gsoli 355 | AB245334.1 | 1495 |
| *Solirubrobacter taibaiensis* GTJR-20 | KF551107.1 | 1387 |
| *Thermoleophilum album* HS-5 | NR_025543.1 | 1460 |
| *Thermoleophilum minutum* ATCC 35268 | NR_036932.1 | 1458 |
